# Real world patterns of dyslipidemia care before and after a national fee revision in Japan: A nationwide study using a 30 million patient claims database

**DOI:** 10.1371/journal.pone.0358270

**Published:** 2026-09-15

**Authors:** Satoshi Yamasaki, Tomotake Tokunou, Takahiko Horiuchi

**Affiliations:** 1 Department of Hematology, St. Mary’s Hospital, Kurume, Japan; 2 Department of Medicine, Fukuoka Dental College, Fukuoka, Japan; 3 Fukuoka City Hospital, Fukuoka, Japan; National Healthcare Group, SINGAPORE

## Abstract

Dyslipidemia is a key risk factor for cardiovascular disease, and its prevalence continues to rise with population aging in Japan. A 2024 change to the fee categories in Japan’s insurance system aimed to encourage a more holistic approach and better patient involvement in developing treatment plans; however, the real-world impact of these changes on patient outcomes remains unclear. Using a nationwide claims database, we sought to: (1) describe the achievement of low-density lipoprotein cholesterol goals and major adverse cardiovascular events among patients with dyslipidemia; (2) compare lipid control and outcomes under the previous disease-specific management fee with those under the new lifestyle disease management fee, and (3) identify patient groups that may benefit from additional lifestyle-based interventions. We conducted a retrospective cohort study among adults aged ≥ 18 years with dyslipidemia (International Classification of Diseases, Tenth Revision code E78) and at least one lipid measurement. In total, 590,000 patients contributed 23,600,010 outpatient visits. Ourpatient visits were the unit of observation for lipid and fee-category analyses; cardiovascular outcomes and patient-years were analyzed at the patient level. The primary outcome was low-density lipoprotein cholesterol goal attainment; secondary outcomes were changes in lipid parameters, major adverse cardiovascular events, and all-cause mortality. Low-density lipoprotein cholesterol goals were attained at 58.3% of outpatient visits under the previous fee and 58.5% under the new fee, and less often in secondary than in primary prevention, despite high statin use and frequent combination therapy with ezetimibe or fibrate. Lipid levels were broadly similar across fee periods. Cardiovascular event rates were also similar, but post-revision patient-years were limited to approximately 6 months (58,500 vs. 1,542,800 patient-years); therefore, this comparison is exploratory and cannot exclude an effect of the new fee structure. Longer follow-up and complementary designs are needed to evaluate the causal impact of the new fee on outcomes.

## Introduction

Dyslipidemia is a major modifiable risk factor for cardiovascular disease, and its prevalence continues to increase in Japan, together with rapid population aging [1]. Despite the widespread use of lipid-lowering agents such as statins, ezetimibe, and proprotein convertase subtilisin/kexin type 9 (PCSK9) inhibitors, real-world data from large administrative databases have consistently shown that a substantial proportion of high-risk patients fail to achieve guideline-recommended low-density lipoprotein cholesterol (LDL-C) targets [2]. Recent real-world observational data from cardiology outpatient clinics in Turkey similarly showed that many patients remain above target despite intensified statin-based therapy, and that adding ezetimibe improved but did not normalize goal attainment [3]. This residual risk highlights the limitations of pharmacotherapy alone and underscores the need for sustainable, lifestyle-based strategies that can be implemented as part of routine care.

Complementary and non-pharmacological approaches, such as structured counseling on diet, physical activity, weight management, and smoking cessation, have attracted growing interest as adjuncts to conventional dyslipidemia management. These lifestyle-based strategies are increasingly embedded in chronic care programs and reimbursement schemes, including Japan’s new lifestyle disease management fee.

The Japanese government has recognized the importance of addressing the social determinants of health in reducing and managing the risk of non-communicable diseases, such as cardiovascular disease, and has made changes to the medical reimbursement system to reflect this [4]. In Japan, most residents are covered by public insurance schemes, and patients generally pay a fixed copayment at the point of care. The remainder is reimbursed directly from insurers to medical facilities based on the national fee schedule. In 2024, the long-standing “specific disease management fee” for conditions such as hypertension, diabetes, and dyslipidemia was replaced by a new “lifestyle disease management fee,” which took effect on 1 June 2024 [5]. These “management fees” are paid to providers as add-on fees for ongoing, structured chronic disease management, rather than as direct payments to patients. The design of the management fee categories may therefore influence provider behavior, adoption, and adherence to chronic care programs. Eligibility for the lifestyle-related disease management fee, which is applicable to outpatient visits only and billed on a per-visit basis, requires physicians to work with patients to create and periodically review a structured Treatment Plan Sheet [5].

This covers both biomedical targets (e.g., LDL-C levels) and broader lifestyle and social determinants (e.g., sleep, physical activity, work conditions, body weight). This reform therefore comprised both a technical change in coding and a broader shift in the institutional framework for chronic disease care. The new management fee emphasizes comprehensive lifestyle management with integrated goals and includes lifestyle counseling, rather than the previous approach in which comorbidities were effectively treated in parallel with standard pharmacotherapy and brief, disease-focused counseling during routine outpatient visits. Pharmacotherapy remains guideline-based and disease-appropriate under the new system. The intended added value lies in the more holistic, goal-oriented, and lifestyle-focused management framework, rather than in changes to drug regimens [6]. Overall, the fee revision was designed to encourage a more comprehensive, collaborative approach, with patients and physicians working together to create and review treatment plans. In theory, this approach should enhance adherence and outcomes. However, as far as can be ascertained, no study to date has examined the effect of these changes on patient outcomes related to dyslipidemia. Therefore, questions remain, such as how often LDL-C goals are achieved in routine care, and whether there have been reductions in major adverse cardiac events.

To address this gap, we conducted a nationwide retrospective cohort study using the Medical Data Vision (MDV) Diagnosis Procedure Combination (DPC) database, which includes approximately 30 million patients across acute care hospitals in Japan. We explored patterns of dyslipidemia care and outcomes before and after the 2024 fee revision to identify gaps in current care and potential target populations for future lifestyle-related interventions. Given the short post-revision observation window and observational design of the present study, our objective was not to estimate the causal effect of the fee revision but to provide an early descriptive snapshot of care patterns and outcomes surrounding implementation of the new lifestyle disease management fee.

## Methods

### Real-world data source

We conducted a retrospective, observational study using the MDV (Medical Data Vision Co., Ltd., Tokyo, Japan) administrative claims and laboratory database, one of the largest hospital-based datasets in Japan. This database covers approximately 30 million patients from more than 450 acute care hospitals participating in the DPC system, including tertiary referral centers and large community hospitals. The MDV database excludes most small clinics and non-DPC facilities. It contains detailed information on demographics, diagnoses (International Classification of Diseases, Tenth Revision codes), procedures, prescriptions, hospitalizations, and laboratory test results. This broad coverage makes the MDV database well-suited for evaluating real-world treatment patterns and outcomes in patients with dyslipidemia. This database was accessed for research purposes on 15 October 2025. The authors had no access to any information that could directly identify individual participants at any time during or after data collection.

### Study design and population

We identified adults (aged ≥ 18 years) with at least one International Classification of Diseases, Tenth Revision code for dyslipidemia (E78.x) and at least one recorded measurement of lipid parameters, total cholesterol (TC), LDL-C, high-density lipoprotein cholesterol (HDL-C), and triglycerides (TG), between 1 January 2021 and 31 December 2024. The index date (baseline) was defined as the earliest date on which both a dyslipidemia diagnosis and a lipid measurement were recorded during the study period. Inclusion criteria were: (1) age ≥ 18 years on the index date; (2) continuous observation in the database for at least 6 months after the index date; and (3) available baseline lipid data. We excluded patients with missing key demographic variables (age or sex) or incomplete baseline lipid data, as well as those with a follow-up duration of < 6 months. To reflect changes in the fee policy, we divided the observation period into two fee categories: (1) the specific disease management fee period (M1), from 1 January 2021–31 May 2024; and (2) the lifestyle disease management fee period (M2), from 1 June 2024–31 December 2024, during which time the new lifestyle disease management fee for hypertension, diabetes, and dyslipidemia was in place. Each patient was classified according to the predominant disease management fee category recorded during follow-up. In sensitivity analyses, we distinguished between patients who were treated for the first time under M2 and those who transitioned from M1 to M2. The primary unit of analysis for baseline characteristics was the patient; for the analyses of fee categories and visit-level outcomes, we used outpatient visits as the unit of observation. Individual patients could contribute multiple visits and could contribute visits to both fee categories, depending on the timing of their follow-up relative to the 2024 fee revision.

### Cohort subgroups

Prespecified subgroups were defined according to: (1) age (< 65, 65–74, and ≥ 75 years); (2) cardiovascular risk category (primary prevention vs. secondary prevention after atherosclerotic cardiovascular disease, and the presence of diabetes mellitus or chronic kidney disease); and (3) baseline lipid levels. These strata were chosen to align with Japanese Atherosclerosis Society (JAS) guidelines [7] and to capture populations most likely to be considered for lifestyle-oriented or complementary interventions in future studies [8]

### Exposures and comparators

The primary exposure was the disease management fee category (M1 vs. M2), reflecting the policy shift from disease-specific to lifestyle disease management for chronic cardiometabolic conditions. Pharmacological treatment patterns for dyslipidemia were also evaluated, including use of statins, ezetimibe, fibrates, PCSK9 inhibitors, and other lipid-lowering agents, alone or in combination. We compared lipid control and cardiovascular outcomes between the M1 and M2 periods overall and within age and risk subgroups, accounting for differences in drug regimens and comorbidities.

### Outcomes

The primary outcome was the attainment of LDL-C goals, defined as achievement of JAS guideline targets based on cardiovascular risk: < 120 mg/dL in primary prevention and < 100 mg/dL (or < 70 mg/dL for very high-risk patients) in secondary prevention. Secondary lipid outcomes included absolute changes in TC, HDL-C, and TG from baseline to follow-up assessments. Clinical secondary outcomes included major adverse cardiovascular events (myocardial infarction, ischemic stroke, hospitalization for heart failure, and cardiovascular death), all-cause mortality, and hospitalization for any cardiovascular cause. We also assessed treatment persistence and adherence to lipid-lowering therapy, as reflected by prescription continuity in the claims data.

For each disease management fee category, on-treatment lipid parameters (TC, LDL-C, HDL-C, and TG) were defined as the most recent lipid measurement obtained during the observation window while the patient was receiving lipid-lowering therapy. LDL-C goal attainment was assessed at this most recent on-treatment measurement under the predominant fee category (M1 or M2). For patients with health care visits during both fee periods, goal attainment was evaluated separately for each period using the relevant on-treatment measurements.

### Statistical analysis

We used descriptive statistics to summarize patients’ baseline characteristics, disease management fee categories, and treatment patterns. We reported the mean with standard deviation or median with interquartile range for continuous variables and the frequency with percentage for categorical variables. For between-group comparisons (e.g., M1 vs. M2, age, and risk strata), we used chi-square tests for categorical variables and the Student *t*-test or analysis of variance for continuous variables. Additionally, we calculated standardized mean differences (SMDs) for baseline characteristics to assess the magnitude of between-group differences independent of the large sample size. An SMD with an absolute value < 0.1 was interpreted as indicating a negligible imbalance, consistent with common practice in observational studies. Cardiovascular outcomes and all-cause mortality were analyzed at the patient level rather than at the visit level. For each fee period, every patient who contributed at least one outpatient visit during that period was followed from the first qualifying visit until the event of interest, loss of database observation, or end of that fee period, whichever occurred first; crude incidence rates were then calculated per 1000 patient-years with exact (Poisson) 95% confidence intervals (CIs). Because a patient could contribute visits to both fee periods, and multiple visits within the same period, the M1 and M2 groups are not independent samples and visit-level proportions are clustered within patients. To avoid overstating precision, 95% CIs for visit-level proportions were calculated using the number of unique patients contributing visits in each stratum as the effective sample size, which is a deliberately conservative approach. Visit-level counts are reported as the number of outpatient visits and are never interpreted as the number of patients. All analyses were descriptive and exploratory; this study was not designed or powered to estimate causal effects of the fee revision. For all statistical analyses, we used EZR (Saitama Medical Center, Jichi Medical University, Saitama, Japan) [9], a graphical user interface for R that extends the functionality of R Commander, as well as R version 4.3.1 (R Foundation for Statistical Computing, Vienna, Austria).

### Ethical considerations

The MDV database contains fully anonymized patient-level data collected for administrative and research purposes. In accordance with Japanese ethical guidelines for medical and health research involving human subjects, the use of anonymized secondary data in this study did not require individual informed consent. The study was approved by the Institutional Review Board of St. Mary’s Hospital, Kurume, Japan (approval number 25–0912).

## Results

### Study population and baseline characteristics

From approximately 30 million patients in the MDV DPC database, we identified 590,000 adults with dyslipidemia who had at least one lipid measurement and at least 6 months of follow-up between 2021 and 2024 (Table 1). Most visits occurred in large acute care hospitals participating in the DPC system, including university hospitals and tertiary centers, with the remainder seen at non-university acute care hospitals. Among the 590,000 patients with dyslipidemia and at least 6 months of follow-up, approximately 472,000 (roughly 80%) had visits only under the specific disease management fee (M1), approximately 38,000 (roughly 6%) had visits only under the lifestyle disease management fee (M2), and approximately 79,000 (roughly 14%) had visits during both periods; this indicated that the M1 and M2 visit counts partially reflected the same patients over time.

**Table 1:** Baseline characteristics of the outpatient cohort with dyslipidemia, by management fee category (unit of observation: outpatient visits).

Variable	Specific disease management fee period (M1)	Lifestyle disease management fee period (M2)
**Total outpatient visits, n**	21,000,013	2,599,997
**Unique patients contributing visits, n**	551,000	117,000
**Age, years**		
Mean ± SD	67.4 ± 12.6	66.9 ± 12.8
Median (IQR)	68 (59–76)	67 (58–75)
**Age groups, n (%)**		
< 65 years	13,100,013 (62.4)	1,719,997 (66.2)
65–74 years	5,899,997 (28.1)	639,999 (24.6)
≥ 75 years	2,000,003 (9.5)	240,001 (9.2)
**Men, n (%)**	11,000,003 (52.4)	1,399,999 (53.8)
**Comorbidities, n (%)**		
Hypertension	14,300,009 (68.1)	1,699,993 (65.4)
Diabetes mellitus	8,199,997 (39.1)	979,997 (37.7)
Prior myocardial infarction	2,599,993 (12.4)	319,991 (12.3)
Prior ischemic stroke	1,899,997 (9.1)	229,993 (8.8)
Prior heart failure	1,749,991 (8.3)	209,989 (8.1)
**Cardiovascular risk category, n (%)**		
Primary prevention	14,499,991 (69.1)	1,779,991 (68.5)
Secondary prevention	6,500,022 (31.1)	820,006 (31.5)
**Baseline lipid parameters, mg/dL**		
Total cholesterol		
Mean ± SD	217.8 ± 38.2	216.6 ± 38.6
Median (IQR)	214 (191–241)	213 (190–240)
LDL-C		
Mean ± SD	138.1 ± 32.4	137.7 ± 32.8
Median (IQR)	135 (115–158)	134 (114–157)
HDL-C		
Mean ± SD	52.6 ± 14.1	52.4 ± 14.3
Median (IQR)	51 (43–61)	50 (42–60)
Triglycerides		
Mean ± SD	155.7 ± 89.1	154.7 ± 89.6
Median (IQR)	137 (97–191)	136 (96–190)
**Current smoker, n (%)**	4,299,997 (20.5)	519,997 (20.1)

Data are presented as n (%) for outpatient visits, unless otherwise specified; the unit of observation is the outpatient visit, and visit counts do not represent counts of distinct patients. The number of unique patients contributing visits in each fee period is shown separately.

Standardized mean difference (M2 vs. M1) values were generally < 0.1, indicating a good balance in baseline characteristics between M1 and M2.

IQR, interquartile range; LDL-C, low-density lipoprotein cholesterol; HDL-C, high-density lipoprotein cholesterol; SD, standard deviation.

The outpatient cohort comprised 23,600,010 visit-level records, of which 21,000,013 visits were reimbursed under the specific disease management fee (M1) and 2,599,997 under the lifestyle disease management fee (M2). Accordingly, 551,000 unique patients (472,000 with M1 visits only plus 79,000 with visits in both periods) contributed the 21,000,013 M1 visits, and 117,000 unique patients (38,000 with M2 visits only plus 79,000 with visits in both periods) contributed the 2,599,997 M2 visits; the M1 and M2 visit counts therefore describe outpatient encounters, not distinct individuals. The mean patient age was similar across categories (67.4 years in M1 and 66.9 years in M2), with roughly one-third of visits involving patients aged 65–74 years and approximately 9%–10% involving those aged ≥ 75 years. Men accounted for just over half of visits in both groups (52.4% in M1 and 53.8% in M2).

Although fewer patients contributed to M2 and its post-fee-revision observation window was shorter, the average number of outpatient visits per patient was higher in M2, resulting in a comparable total number of visits across the two fee categories over the study period. Each outpatient visit was treated as a separate observation, and individual patients could contribute multiple visits to either fee category depending on the timing of their follow-up relative to the 2024 fee revision. Comorbidities were common: hypertension was recorded in 68.1% of M1 visits and 65.4% of M2 visits and diabetes mellitus in 39.1% and 37.7%, respectively. Prior cardiovascular disease was also frequent, with 12.4% versus 12.3% of visits associated with previous myocardial infarction, 9.1% versus 8.8% with previous ischemic stroke, and 8.3% versus 8.1% with previous heart failure in M1 and M2.

The distribution of cardiovascular risk categories was nearly identical between fee categories, with primary prevention accounting for 69.1% of M1 visits, 68.5% of M2 visits, and secondary prevention for 31.1% and 31.5%, respectively. Baseline lipid profiles were also highly comparable: mean TC was 217.8 mg/dL in M1 and 216.6 mg/dL in M2, mean LDL-C was 138.1 versus 137.7 mg/dL, mean HDL-C was 52.6 versus 52.4 mg/dL, and mean TG was 155.7 versus 154.7 mg/dL, with similar median and interquartile range values. Current smoking was reported in approximately one-fifth of visits (20.5% in M1 and 20.1% in M2). Across these variables, SMDs were generally below 0.1, indicating a good balance in baseline demographic, clinical, and lipid characteristics between M1 and M2, despite the large sample size.

### Pharmacological lipid-lowering therapy and on-treatment lipid levels

During both study periods, most outpatient visits involved active pharmacological management of dyslipidemia (Table 2). Statins were prescribed in 81.9% of M1 visits and 82.3% of M2 visits; ezetimibe in 27.6% and 27.7%, respectively; fibrates in 11.9% of visits in both groups; and PCSK9 inhibitors in 2.5% of visits in each category. On-treatment lipid profiles were modestly improved as compared with baseline, but substantial residual dyslipidemia persisted.

**Table 2:** Lipid profile and treatment patterns by management fee category.

Parameter	Specific disease management fee period (M1) (21,000,013 outpatient visits)	Lifestyle disease management fee period (M2) (2,599,997 outpatient visits)
**Lipid-lowering therapy, n (%)**		
Statin use	17,199,997 (81.9)	2,139,991 (82.3)
Ezetimibe use	5,799,991 (27.6)	719,993 (27.7)
Fibrate use	2,499,997 (11.9)	309,989 (11.9)
PCSK9 inhibitor use	519,991 (2.5)	63,997 (2.5)
		
**Lipid parameters (on treatment), mg/dL**		
Total cholesterol		
Mean ± SD	195.5 ± 36.0	194.9 ± 36.3
Median (IQR)	191 (171–217)	190 (170–216)
LDL-C		
Mean ± SD	114.8 ± 30.3	114.3 ± 30.6
Median (IQR)	111 (93–133)	110 (92–132)
HDL-C		
Mean ± SD	54.8 ± 14.7	54.4 ± 14.9
Median (IQR)	53 (45–63)	52 (44–62)
Triglycerides		
Mean ± SD	141.8 ± 82.0	140.9 ± 82.5
Median (IQR)	125 (88–177)	124 (87–176)
**LDL-C goal attainment** ^**a**^ **, n (%)**		
Overall cohort	12,249,996 (58.3)	1,519,998 (58.5)
Primary prevention group	8,620,003 (59.4)	1,069,999 (60.1)
Secondary prevention group	3,629,993 (55.8)	449,999 (54.9)

^a^LDL-C goals based on Japanese Atherosclerosis Society guidelines: < 120 mg/dL in primary prevention, < 100 mg/dL (or < 70 mg/dL for very high-risk) in secondary prevention.

Data are shown as n (%) or mean ± SD.

Values represent the most recent on-treatment lipid measurement within each patient’s observation period under the corresponding management fee category. Data are shown per outpatient visit as the unit of analysis because the lifestyle disease management fee is applicable only in the outpatient setting and on a per-visit basis.

LDL-C, low-density lipoprotein cholesterol; HDL-C, high-density lipoprotein cholesterol; IQR, interquartile range; PCSK9, proprotein convertase subtilisin/kexin type 9; SD, standard deviation.

Mean TC declined to 195.5 mg/dL in M1 and 194.9 mg/dL in M2, and mean LDL-C dropped to 114.8 mg/dL and 114.3 mg/dL, respectively. Mean HDL-C increased slightly to 54.8 mg/dL in M1 and 54.4 mg/dL in M2, whereas mean TG decreased to 141.8 mg/dL and 140.9 mg/dL, respectively. Differences in on-treatment lipid parameters between M1 and M2 were small and not clinically meaningful; SMDs were < 0.10 for all lipid variables, and no statistically significant differences were detected using *t*-*t*ests or analysis of variance after adjustment for the large sample size. These findings suggest that the initial transition from specific disease management fee (M1) to lifestyle disease management fee (M2) did not substantially alter visit-level treatment intensity or on-treatment lipid levels within the observation window.

### LDL-C goal attainment overall and by prevention category

Despite intensive pharmacotherapy at the visit level, guideline-recommended LDL-C goals based on JAS criteria were achieved in only approximately 60% of visits. Overall, LDL-C targets were attained in 58.3% of M1 visits and 58.5% of M2 visits. The difference between M1 and M2 was statistically negligible (chi-square test, p < 0.05; SMDs < 0.01), consistent with very similar patterns of pharmacological management across fee categories. LDL-C goal attainment was modestly higher in primary prevention visits (59.4% in M1 and 60.1% in M2) than in secondary prevention visits (55.8% and 54.9%, respectively), suggesting considerable residual risk, particularly among patients with established atherosclerotic cardiovascular disease. Within each prevention stratum, however, the differences between M1 and M2 were again small, with overlapping 95% CIs and SMDs < 0.05.

### Age-stratified LDL-C goal attainment and impact of fee transition

**Fig 1:**
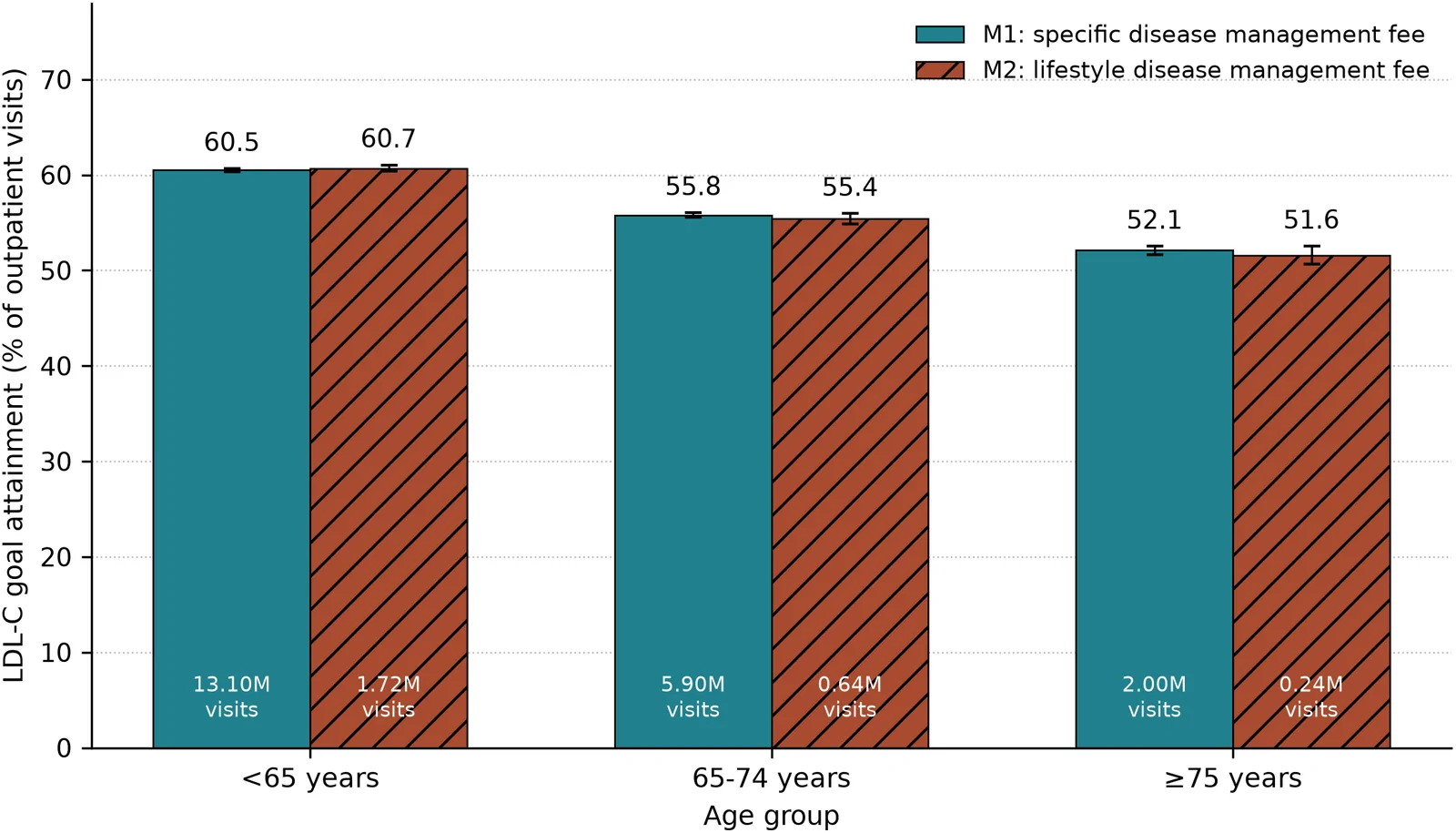
Low-density lipoprotein cholesterol (LDL-C) goal attainment by age and management fee category. Age-stratified LDL-C goal attainment among outpatient visits under the specific disease management fee (M1) and lifestyle disease management fee (M2).Each bar represents the proportion of outpatient visits at which patients had achieved the LDL-C target, stratified by age group and fee category. A total of 21,000,013 outpatient visits (from 551,000 unique patients) were classified as M1 and 2,599,997 visits (from 117,000 unique patients) as M2; the numbers shown within the bars are outpatient visits, not individual patients. The higher number of M1 visits primarily reflects the longer pre-revision observation period and the inclusion of patients already under follow-up prior to the 2024 revision, rather than a systematically higher visit frequency per patient. Error bars are 95% confidence intervals calculated using the number of unique patients contributing visits in each stratum as the effective sample size, so as to account for clustering of repeated visits for the same patient.

Across 23,600,010 outpatient visits, LDL-C goal attainment declined progressively with advancing age under both fee categories. The lowest attainment rates were observed among visits involving patients aged ≥ 75 years in both M1 and M2 (Fig 1). In age-stratified analyses (< 65, 65–74, and ≥ 75 years), LDL-C goal attainment was consistently highest in those aged < 65 years, lower in those aged 65–74 years, and lowest in those aged ≥ 75 years, reflecting the greater burden of comorbidities and more refractory dyslipidemia in older patients.

In each age stratum, the difference in LDL-C goal attainment between M1 and M2 was small, with overlapping 95% CIs and odds ratios close to 1.0 (all 95% CIs included 1.0). These age-stratified comparisons were tested using chi-square tests and supplemented with SMDs, which were uniformly < 0.10, indicating no material imbalance in visit-level LDL-C control between fee categories. Taken together, the similarity in lipid-lowering treatment patterns, on-treatment lipid levels, and age-stratified LDL-C goal attainment between M1 and M2 suggests that, within the short follow-up period after the 2024 fee revision, the transition from specific disease management fee to lifestyle disease management fee did not lead to detectable improvements in LDL-C control at the visit level.

### Major adverse cardiovascular events

Cardiovascular outcomes were assessed at the patient level (Table 3). The 551,000 patients who contributed M1 visits were followed for a mean of 2.8 years, yielding 1,542,800 patient-years of observation. Because the lifestyle disease management fee was introduced on 1 June 2024, the 117,000 patients who contributed M2 visits could be followed for a mean of only 0.5 years, yielding 58,500 patient-years, approximately 26-fold fewer patient-years than in M1. Incidence rates of major adverse cardiovascular events were substantial and numerically similar between fee periods. For the composite of myocardial infarction, ischemic stroke, and cardiovascular death, there were 42,242 events in M1 (7.7% of M1 patients) and 1591 events in M2 (1.4% of M2 patients), corresponding to incidence rates of 27.38 (95% CI 27.12–27.64) and 27.20 (95% CI 25.88–28.57) per 1000 patient-years, respectively. Given the markedly unequal and very short post-revision observation window, this similarity should be regarded as descriptive only and must not be read as evidence that the fee revision had no effect on cardiovascular outcomes.

**Table 3:** Patient-level major adverse cardiovascular events in the study population, by management fee period.

Outcome	M1: No. of patients with event	M1: Incidence rate per 1000 patient-years (95% CI)	M2: No. of patients with event	M2: Incidence rate per 1000 patient-years (95% CI)
**Follow-up duration** ^**a**^				
Mean follow-up, years	2.8	—	0.5	—
Total patient-years	1,542,800	—	58,500	—
**Cardiovascular outcomes**				
All cardiac events^b^	42,242	27.38 (27.12–27.64)	1,591	27.20 (25.88–28.57)
All-cause mortality	25,194	16.33 (16.13–16.53)	948	16.21 (15.19–17.27)
Myocardial infarction	15,474	10.03 (9.87–10.19)	587	10.03 (9.24–10.88)
Ischemic stroke	12,065	7.82 (7.68–7.96)	466	7.97 (7.26–8.72)
Hospitalization for heart failure	16,786	10.88 (10.72–11.05)	659	11.26 (10.42–12.16)

^a^ All cardiac events includes myocardial infarction, ischemic stroke, and cardiovascular death.

^b^ Events, patient-years, and incidence rates are at patient level: the denominators are 551,000 patients (1,542,800 patient-years) for M1 and 117,000 patients (58,500 patient-years) for M2, not outpatient visits. Because the lifestyle disease management fee took effect on 1 June 2024, the maximum post-implementation follow-up was 7 months (mean 0.5 years); thus, approximately 26-fold fewer patient-years were accumulated in M2 than in M1. Rates for M2 are therefore imprecise and are not comparable to those for M1 without strong assumptions; the numerical similarity of rates must not be interpreted as evidence the fee revision had no effect.

M1, specific disease management fee period; M2, lifestyle disease management fee period; CI, confidence interval.

All-cause mortality also occurred at similar rates in both periods (16.33 vs. 16.21 per 1000 patient-years; 25,194 deaths in M1 and 948 in M2), with overlapping 95% CIs. Myocardial infarction and ischemic stroke each contributed materially to the event burden, with incidence rates of 10.03 per 1000 patient-years for myocardial infarction in both periods (15,474 vs. 587 events) and 7.82 versus 7.97 per 1000 patient-years for ischemic stroke in M1 and M2, respectively (12,065 vs. 466 events). Hospitalization for heart failure was frequent, with incidence rates of 10.88 per 1000 patient-years in M1 and 11.26 per 1000 patient-years in M2 (16,786 vs. 659 events). These consistently high rates of cardiovascular events and mortality, despite high statin use and broadly similar management under M1 and M2, underscore a substantial residual risk that appears not to have been mitigated during the initial implementation period of the lifestyle disease management fee, suggesting the need for more comprehensive risk-reduction strategies extending beyond pharmacotherapy alone.

## Discussion

This nationwide real-world analysis of 590,000 adults with dyslipidemia who contributed 23.6 million outpatient visits in Japanese acute care hospitals showed that LDL-C goal attainment remains suboptimal at the visit level, despite widespread use of statins and combination lipid-lowering therapy, and that this pattern persisted after the 2024 fee schedule revision. Suboptimal lipid control was evident before this change, and the initial post-implementation period showed no clear improvements in LDL-C goal attainment or in major acute cardiovascular events in this observational setting. The present findings should be interpreted with caution, given the limited post-implementation follow-up and potential for residual confounding. However, these findings suggest that measures beyond fee revision alone will be needed to close the gap between guideline recommendations and routine care. This is broadly consistent with prior Japanese and international reports documenting persistent underachievement of LDL-C targets among high-risk patients [10,11].

The 2024 fee structure was introduced to support more holistic disease management, including explicit attention to lifestyle and social factors, and to promote patient involvement in treatment planning [5]. In principle, such changes could enhance adherence and improve outcomes. However, across the 23.6 million outpatient visits contributed by these patients, JAS LDL-C targets were met at only approximately 58% of visits, with consistently lower attainment in secondary than in primary prevention. These results underscore a substantial residual cardiovascular risk that is unlikely to be mitigated by pharmacotherapy alone and highlight the need for scalable lifestyle-oriented strategies that can be embedded in routine care [4,8,11,12]. Our findings are also consistent with contemporary real-world evidence from other health systems, in which a large share of patients attending specialist outpatient clinics remained above target despite intensification of statin-based regimens [3].

The baseline characteristics in this study illustrate both the scale of dyslipidemia care and the complexity of comorbidity in an aging society. Patients under both fee schemes had a mean age of approximately 67 years; two-thirds of visits involved coexisting hypertension, nearly 40% involved diabetes mellitus, and approximately one-third involved secondary prevention. Despite broadly similar risk profiles between M1 and M2, on-treatment lipid levels remained far from optimal, with mean LDL-C levels of approximately 115 mg/dL and more than 40% of visits failing to meet JAS LDL-C goals (Table 2). Statins were prescribed in more than 80% of visits, and ezetimibe or fibrates were frequently added, indicating that many clinicians had already escalated patients’ pharmacologic regimens. Nevertheless, a large fraction of patients remained above the target, a pattern consistent with claims-based studies from Japan and other countries [13–15].

Cardiovascular outcomes were similarly sobering. Patient-level incidence rates of major acute cardiac events and all-cause mortality were high and numerically comparable between M1 and M2, with approximately 27 events per 1000 patient-years for major acute cardiac events and approximately 11 hospitalizations for heart failure per 1000 patient-years in both periods. These event rates, observed at the patient level over a mean follow-up of 2.8 years and approximately 1.54 million patient-years of observation in M1, underscore that contemporary drug-centered dyslipidemia management has not eliminated the cardiovascular risk in this high-risk population. Importantly, no measurable improvement in LDL-C control was detected after the transition to M2 in mid-2024; for cardiovascular events, only 58,500 patient-years were accumulated between June and December 2024, which was insufficient to detect a change in event rates; thus, the absence of an observed difference is uninformative rather than reassuring.

Several factors may explain the apparent lack of early improvement in our study. First, there was only a brief post-implementation period between the introduction of fee changes in June 2024 and the end of follow-up in December 2024; such a practice change typically requires more time to become embedded. For example, implementation studies of evidence-based practices have reported substantial increases in uptake between 7–9 months and 2.5 years after initial introduction, a time frame that extends beyond our observation window [16]. Second, multiple barriers at the patient, professional, and organizational levels may impede effective implementation of shared, lifestyle-focused care. A systematic review of shared decision-making in older adults with multimorbidity found that poor health status and cognitive or physical impairments reduced patient participation; time constraints and limited communication skills among health care professionals also hindered shared decision-making [17]. Other studies suggest that older adults with a greater burden of health problems may be less likely to favor or engage in shared decision making [18]. At the organizational level, constraints on staff time, workload, and competing priorities in busy outpatient settings likely make structured lifestyle counseling difficult to deliver consistently [17]. Lastly, the financial incentive associated with the lifestyle disease management fee may have been insufficient to offset the additional workload and logistic complexity required to implement structured counseling. Because these mechanisms cannot be disentangled using claims data alone, our findings should be regarded as hypothesis generating. Nevertheless, longer-term follow-up remains important, given the accumulating evidence that structured lifestyle-based interventions can reduce cardiovascular risk in high-risk populations [19–21].

Our results will help to identify groups that can be prioritized for additional support and/or lifestyle interventions. In our study, LDL-C goal attainment declined with advancing age, and patients in secondary prevention were less likely to reach targets than those in primary prevention. Therefore, older adults and patients with prior cardiovascular events appear particularly vulnerable to residual risk despite pharmacotherapy and may benefit most from tailored lifestyle interventions [18]. Paradoxically, these same groups are among those less likely to actively participate in health care decisions, underscoring the need for proactive, patient-centered approaches versus reliance on patient-initiated engagement alone.

In the above context, complementary and non-pharmacological approaches have attracted increasing interest as adjuncts to conventional dyslipidemia management [22]. For example, a recent systematic review of seven randomized controlled trials reported that hot spring or sauna interventions, particularly when combined with exercise, produced modest but clinically relevant reductions in TC and LDL-C among younger or middle-aged adults (mean age < 60–65 years) whereas the effects were small or non-significant in older or multimorbid cohorts [23]. Although such interventions cannot be evaluated directly using claims-based data, they illustrate the potential for structured, context-specific lifestyle programs to complement drug therapy, particularly in settings where thermal and spa resources are readily available.

This study has several strengths. We leveraged one of the largest hospital-based claims and laboratory datasets in Japan, capturing 23.6 million outpatient visits from 590,000 patients and providing a large volume of observations with which to describe LDL-C control across age, risk, and fee strata. The analysis spans both pre- and early post-2024 reimbursement regimes, offering an initial view of how the new lifestyle disease management fee aligns with emerging lifestyle-oriented strategies. However, for detecting changes in clinical practice and outcomes, the post-implementation period is relatively short, and a lag between policy changes and frontline counseling practices is likely. Therefore, we cannot rule out the possibility that longer follow-up will reveal differences in LDL-C control or cardiovascular event rates between M1 and M2. Future analyses should incorporate more recent data not available at the time of this study.

Several limitations should also be considered. The MDV database primarily includes DPC-participating acute care hospitals and may underrepresent care in smaller clinics or non-DPC settings, potentially limiting generalizability of the findings. The database does not capture detailed information on lifestyle behaviors or non-pharmacological interventions, including diet, physical activity, structured counseling, or complementary approaches such as hot spring or sauna therapy. Therefore, we could not evaluate how the fee revision influenced lifestyle-related care processes and could not disentangle the effects of pharmacologic intensification from behavioral change. Residual confounding is inevitable in observational claims analyses; we lacked data on diet, physical activity, sleep, and other lifestyle factors that influence lipid profiles and cardiovascular risk. Some misclassification of fee category during the early implementation phase is also possible. Overall, the present analysis should be viewed as an early snapshot of real-world practice patterns and outcomes surrounding the fee revision, rather than a definitive policy evaluation of the lifestyle disease management fee.

Several further limitations follow directly from the study design and data source. First, the M1 and M2 periods are neither independent nor of comparable length: M1 spans January 2021 to May 2024 (41 months) whereas M2 spans only June to December 2024 (7 months), and 79,000 patients (13% of the cohort) contributed observations to both periods. The M1 versus M2 contrasts are therefore repeated cross-sectional descriptions of the same evolving population rather than comparisons of independent groups, and they remain vulnerable to secular trends, calendar-time effects, seasonal variation in lipid testing, and within-patient correlation of repeated visits. We deliberately refrained from formal hypothesis testing of policy effects for this reason, reported conservative CIs based on the number of unique patients, and interpreted all M1 versus M2 differences as descriptive. Second, the cardiovascular outcome comparison is intrinsically limited by the accumulation of only 58,500 patient-years after implementation; a difference in event rates could not have been detected within this window, even if one had existed, and our findings should not be read as evidence of no effect. Third, the MDV database is built on DPC-participating acute care hospitals and therefore excludes most small clinics, primary care practices, and other non-DPC facilities, where a large share of routine dyslipidemia care and much of the uptake of the lifestyle disease management fee is likely to occur. The cohort is consequently enriched for older, multimorbid, hospital-managed patients, and the findings may not generalize to the overall Japanese population with dyslipidemia. Fourth, the central components of the new management strategy including structured lifestyle counseling, shared goal setting recorded on the Treatment Plan Sheet, medication adherence, dietary modification, physical activity, sleep, smoking behavior, and body weight are not directly measurable using administrative claims. Adherence could only be approximated from prescription continuity, and the delivery or quality of counseling could not be observed at all. Fifth, laboratory results are reported only for tests performed and recorded at participating institutions, so lipid values may not be missing at random, and coding of the fee category during the early implementation phase may be subject to misclassification.

From a clinical and health policy perspective, the 2024 shift to a lifestyle disease management fee represents a timely institutional opportunity to integrate structured lifestyle programs, including exercise, nutrition, sleep counseling, and appropriate complementary thermal interventions into dyslipidemia care. Our findings suggest that any impact of the fee change on clinical outcomes may take time to emerge and may be difficult to detect using routine claims data alone. Future research with longer follow-up, more granular clinical and lifestyle information, and quasi-experimental or pragmatic trial designs, where feasible, will be needed to more rigorously evaluate the long-term effects of the new fee structure. Pragmatic cluster-randomized or stepped-edge trials embedded in M2-based care pathways could test whether adding specific lifestyle or complementary interventions can yield incremental improvements in LDL-C control and major acute cardiac events beyond drug therapy alone. By explicitly linking reimbursement structures, real-world risk profiles, and evidence-based non-pharmacological options, such studies could inform future guideline and policy development for the management of dyslipidemia in aging societies.
