# Supplementary material for: Real world patterns of dyslipidemia care before and after a national fee revision in Japan: A nationwide study using a 30 million patient claims database
Source: PLoS One. 2026 Sep 15;21(9):e0358270. doi: 10.1371/journal.pone.0358270 (PMC13577349; doi:10.1371/journal.pone.0358270)
Supplement: Supplementary file 1 [file pone.0358270.s002.docx]

25 June 2026

Dr. Jennifer Tucker, PhD
Staff Editor
*PLOS One*

Dear Dr. Tucker,

On behalf of my co-authors, I would like to thank you and the reviewers for the thoughtful and constructive comments on our manuscript entitled “Real‑world patterns of dyslipidemia care before and after a national fee revision in Japan: a nationwide study using a 30‑million‑patient claims database” (Manuscript ID: PONE‑D‑26‑19373).

We have carefully revised the manuscript to address all points raised by the reviewers and to align it with *PLOS One*’s style and policy requirements.
We have revised our manuscript in response to these comments, with the updated text highlighted in red. Our detailed point-by-point responses are provided on the following pages.

We hope that the revised version is now suitable for publication in *PLOS One*.

Sincerely,

Satoshi Yamasaki, MD, PhD

Department of Hematology, St. Mary’s Hospital

422 Tsubukuhonmachi, Kurume, Fukuoka 830-8543, Japan

Tel: +81-942-35-3322

Fax: +81-942-34-3115

E-mail: [yamas009@gmail.com](mailto:yamas009@gmail.com)

**POINT-BY-POINT RESPONSES TO THE REVIEWERS’ COMMENTS**

**Reviewers’ Comments:
Reviewer #1 (Comments to the Author (Required)):
Very good study using large national databases. However, few improvements are needed especially in the method and result section to describe exactly what the study findings can offer.**

**Response:** We sincerely appreciate your positive evaluation and helpful suggestions.
We have revised the Methods and Results sections to clearly define the study population, the units of analysis, the statistical methods used, and the descriptive nature of our study and study aims. We have also modified the title and Introduction to emphasize that this is an early descriptive snapshot rather than a definitive policy evaluation.

**Comment 1**

***To use full term whenever the word is first written, for example, PCSK9 inhibitors.***

**Response:** We have ensured that all abbreviations are spelled out at their first use, including “PCSK9 inhibitors” (Page 4, Line 41).

**Comment 2**

***Confusion of terms involving exact number of patients, where different terms were used (patients, outpatient visits, person-years). Consistent use of terms or defining the terms clearly would be helpful to understand the whole study. And how many are actually transitioned from M1 to M2? Are those numbers reflecting similar patients?***

**Response:** Thank you very much for pointing out these important issues.

In the revised manuscript, we have clarified the main unit of analysis (patient‑level vs. visit-level) and have consistently distinguished “patients” from “outpatient visits” throughout the Methods and Results sections. We added explicit definitions of these terms in the Methods section (Page 9, Lines 127–132) and summarized the number of patients who transitioned from M1 to M2, clarifying that some individuals contributed visits to both fee categories (Page 13, Lines 202–210). In addition, we have revised ambiguous expressions in the Results, replacing “patients” with “visits” when referring to visit‑based denominators. Regarding the proportions of patients in each fee category, the underlying denominators for each group are slightly rounded, and the exact percentages cannot be determined with high precision. To avoid over‑interpretation, we have therefore retained the wording “approximately” and rounded the proportions to whole numbers (80%, 6%, and 14%), while clearly indicating that some patients are represented in both M1 and M2 over time.

**Comment 3**

***The discussion section elaborates on the lifestyle-related care processes, including diet, physical activity etc, while the introduction part mentions sauna and hot spring interventions. Suggest to omit and replace with the lifestyle adjustments discussed in the discussion section.***

**Response:**
We appreciate this suggestion and agree that the Introduction should align with the lifestyle-related care processes directly relevant to our analysis.
We have therefore shortened the paragraph mentioning sauna and hot spring interventions and have refocused the Introduction on lifestyle counseling within routine dyslipidemia care (diet, physical activity, weight management, smoking cessation), which is embedded in the new lifestyle disease management fee (Page 4, Lines 47−51). We removed the detailed description of hot spring/sauna RCTs from the Introduction and retained only a brief mention in the Discussion when describing potential lifestyle‑based interventions for selected subgroups, to keep the focus on care processes that can be routinely implemented in outpatient settings.

**Comment 4**

***“Overall, the present analysis should be regarded as an early snapshot of real-world practice patterns surrounding the fee revision, rather than a definitive policy evaluation of the lifestyle disease management fee.” -- I agree with what is being written in the discussion where this study analyzes the findings descriptively rather than an impact of policy. The whole manuscript then can be rewritten especially the title, introduction, and method, to not highlight the impact of policy changes.***

**Response:**
Thank you for this important clarification.
We fully agree that our study is descriptive and that causal policy effects cannot be inferred from this early observational analysis. To reflect this, we have revised the title, Abstract (Page 2, Lines 17−22; Page 3, Lines 32−35), Introduction (Pages 6−7, Lines 88−91), and Methods (Page 12, Lines 181−184) to consistently emphasize the descriptive, early “snapshot” nature of our work rather than a definitive policy impact evaluation.

**Comment 5**

***The figure legend is too long; it should be described in the result section.***

**Response:**
We agree that the figure legend was overly detailed.
We have shortened the figure legend and retained only essential information (Pages 37−38, Lines 611−619). We have moved the more detailed description of age‑stratified LDL‑C goal attainment to the Results section (Pages 16−21, Lines 247−311).

**Comment 6**

***To revise the statistical analysis part in the method section, as it is not tailored to what being written in the results. (Mixed-effects models, Cox proportional hazards models, Adjustment for covariates, Sensitivity analyses all are mentioned, but not displayed in the result section.)***

**Response:**
Thank you for highlighting this mismatch.
In the current revision, we chose to focus the manuscript on the descriptive analyses, per your earlier comment (baseline characteristics, treatment patterns, LDL‑C goal attainment, and crude event rates), which are presented in the Results. We therefore removed the description of mixed‑effects models and Cox proportional hazards models from the Methods and clarified that our comparisons are primarily descriptive.

**Reviewer #2 (Comments to the Author (Required)):
Comment 1 – Title**

***The current title uses repetitive words ("disease" appears 4 times, "management" appears twice). Authors should consider revising the title to avoid these repetitions.***

**Response:**
We appreciate this suggestion and have revised the title to avoid repetition and emphasize the study's descriptive nature.

**Comment 2 – Abbreviations**

***In the introduction, some abbreviations not initially defined e.g. PCSK9.***

**Response:** We agree and have ensured that all abbreviations are spelled out at first use, including “PCSK9 inhibitors” (Page 4, Line 41).

**Comment 3 – Study population and criteria**

***In the methods, authors should clearly define the study population and selection (inclusion and exclusion) criteria.***

**Response:** Thank you for this important point. We have expanded the “Study design and population” subsection to explicitly list the inclusion and exclusion criteria and have clarified the index date definition and minimum follow‑up period (Page 8, Lines 116−120).

**Comment 4 – Hospital type**

***In the results section, authors should indicate the type/category of hospitals where patients were receiving care.***

**Response:**
We agree that specifying hospital types helps readers understand generalizability.
We have added a description of the hospital categories in the Methods section (Page 7, Lines 99-101) and summarized their distribution in the Results section (Page 13, Lines 202−204).

**Comment 5 – Statistical comparisons and time series**

***Statistical comparison using t-tests, odds ratios, and the time series plot is not seen in the results.***

**Response:**
We thank the reviewer for this important observation.

In the revised manuscript, we have clarified and aligned the statistical methods with the analyses presented in the Results. Specifically, we now explicitly state in the Methods that we used chi‑square tests for categorical variables, *t*‑tests or ANOVA for continuous variables, and standardized mean differences to evaluate the magnitude of differences between fee categories, rather than fitting mixed‑effects or Cox models.

In the Results, we now report these comparisons more transparently. In the section “Pharmacological lipid‑lowering therapy and on‑treatment lipid levels,” we note that between‑group differences in on‑treatment lipid parameters were small (all standardized mean differences < 0.10) and not clinically meaningful, and that no statistically significant differences were detected by *t*‑tests or ANOVA after accounting for the large sample size (Pages 16–18, Lines 247–278). In the sections “LDL‑C goal attainment overall and by prevention category” and “Age‑stratified LDL‑C goal attainment and impact of fee transition,” we now explicitly report chi‑square p‑values, standardized differences, and age‑stratified odds ratios with 95% confidence intervals, showing that the differences between M1 and M2 were uniformly small and that all confidence intervals overlapped 1.0 (Pages 18–20, Lines 280–311).

Regarding the time‑series aspect, our primary unit of analysis in this study is the outpatient visit rather than the individual patient, and the current manuscript focuses on cross‑sectional before‑and‑after comparisons (pre‑ vs. post‑revision fee categories) and age‑stratified patterns. We therefore present an updated Figure 1 that depicts age‑stratified LDL‑C goal attainment for M1 and M2, rather than a patient‑level longitudinal time‑series model (Pages 19–20, Lines 293–311). We have removed the description of mixed‑effects and Cox proportional hazards models from the Methods to avoid mentioning analyses that are not directly reflected in the Results. We have also clarified that more complex longitudinal and time‑series modeling should be addressed in a separate, methodologically focused report.

**Response to cover letter (Journal Requirements):**

We appreciate the Editor’s guidance regarding the Funding Information and Financial Disclosure sections.
In the revised submission, we have ensured that the funding information is consistent and restricted to the Funding Statement in the online submission form.
Specifically, we have provided the following funding and role of the funder statement:

“This work was supported by the Health, Labour, and Welfare Scientific Research Fund (grant no. 24FA1004). The funders had no role in the study design, data collection and analysis, decision to publish, or preparation of the manuscript. The authors funded the article processing.”

We have removed funding information from the manuscript text, including the Acknowledgments and any other sections, in accordance with *PLOS One* policy.

**Ethics statement**

**Response to cover letter (Journal Requirements):**

We have revised the manuscript such that the ethics statement appears only in the Methods section. Any repeated ethics statements in other sections have been removed.

**Data availability**

**Response to cover letter (Journal Requirements):**

“The data underlying this study are third‑party claims data from the Medical Data Vision (MDV) database in Japan. The authors are not permitted to publicly share raw data because of contractual and legal restrictions related to patient privacy. Researchers who meet the criteria for access to confidential data can apply directly to Medical Data Vision Co., Ltd. (https://www.mdv.co.jp) for data access under a data use agreement, with approval from their institutional ethics committee. The authors did not have any special access privileges that others did not have.” (Pages 30–31, Lines 490–503)
