# Supplementary material for: Real world patterns of dyslipidemia care before and after a national fee revision in Japan: A nationwide study using a 30 million patient claims database
Source: PLoS One. 2026 Sep 15;21(9):e0358270. doi: 10.1371/journal.pone.0358270 (PMC13577349; doi:10.1371/journal.pone.0358270)
Supplement: Supplementary file 2 [file pone.0358270.s003.docx]

28 August 2026

Dr. Yee Gary Ang, MBBS MPH
Academic Editor
*PLOS One*

Dear Dr. Ang,

On behalf of my co-authors, I would like to thank you and the reviewers for your thoughtful and constructive comments on our manuscript entitled “Real‑world patterns of dyslipidemia care before and after a national fee revision in Japan: a nationwide study using a 30‑million‑patient claims database” (Manuscript ID: PONE-D-26-19373R1).

We have carefully revised the manuscript to address all points raised by the reviewers and to align it with *PLOS One*’s style and policy requirements.
We have revised our manuscript in response to these comments, with the updated text highlighted in red. Our detailed point-by-point responses are provided below.

We hope that the revised version is now suitable for publication in *PLOS One*.

Sincerely,

Satoshi Yamasaki, MD, PhD

Department of Hematology, St. Mary’s Hospital

422 Tsubukuhonmachi, Kurume, Fukuoka 830-8543, Japan

Tel: +81-942-35-3322

Fax: +81-942-34-3115

E-mail: [yamas009@gmail.com](mailto:yamas009@gmail.com)

**POINT-BY-POINT RESPONSES TO THE REVIEWERS’ COMMENTS**

**Additional Editor Comments:**

**We have invited multiple reviewers.**

**One has accepted but the other still has major comments**

**Reviewers' comments:**

**Reviewer's Responses to Questions**

**Comments to the Author

1. If the authors have adequately addressed your comments raised in a previous round of review and you feel that this manuscript is now acceptable for publication, you may indicate that here to bypass the “Comments to the Author” section, enter your conflict of interest statement in the “Confidential to Editor” section, and submit your "Accept" recommendation.**

**Reviewer #1: All comments have been addressed**

**Reviewer #3: All comments have been addressed**

**2. Is the manuscript technically sound, and do the data support the conclusions?

The manuscript must describe a technically sound piece of scientific research with data that supports the conclusions. Experiments must have been conducted rigorously, with appropriate controls, replication, and sample sizes. The conclusions must be drawn appropriately based on the data presented.**

**Reviewer #1: Yes**

**Reviewer #3: Yes**

**3. Has the statistical analysis been performed appropriately and rigorously?**

**Reviewer #1: Yes**

**Reviewer #3: Yes**

**4. Have the authors made all data underlying the findings in their manuscript fully available?

The** [**PLOS Data policy**](http://track.editorialmanager.com/CL0/http:%2F%2Fwww.plosone.org%2Fstatic%2Fpolicies.action%23sharing/1/010f01a03686b21b-a89338d0-4e82-4e4d-ad00-2a28b0d445d2-000000/horhjlJheNu-4szlqgQ13PUQdlHD_kIbbickfrlawt8=258) **requires authors to make all data underlying the findings described in their manuscript fully available without restriction, with rare exception (please refer to the Data Availability Statement in the manuscript PDF file). The data should be provided as part of the manuscript or its supporting information, or deposited to a public repository. For example, in addition to summary statistics, the data points behind means, medians and variance measures should be available. If there are restrictions on publicly sharing data—e.g. participant privacy or use of data from a third party—those must be specified.**

**Reviewer #1: Yes**

**Reviewer #3: Yes**

**5. Is the manuscript presented in an intelligible fashion and written in standard English?

PLOS ONE does not copyedit accepted manuscripts, so the language in submitted articles must be clear, correct, and unambiguous. Any typographical or grammatical errors should be corrected at revision, so please note any specific errors here.**

**Reviewer #1: Yes**

**Reviewer #3: Yes**

**6. Review Comments to the Author

Please use the space provided to explain your answers to the questions above. You may also include additional comments for the author, including concerns about dual publication, research ethics, or publication ethics. (Please upload your review as an attachment if it exceeds 20,000 characters)**

**Reviewer #1: (No Response)**

**Reviewer #3: The revised manuscript addresses several of the previous concerns, and the clarification of the descriptive nature of the study is helpful. The large nationwide dataset is a major strength. However, several important methodological and reporting issues remain and should be addressed before publication.**

**Major concern regarding the cardiovascular outcome denominators.
1.The reported person-years and event numbers appear internally inconsistent with the cohort size. The manuscript reports approximately 590,000 patients, but 58,800,036 person-years in M1 with a mean follow-up of 2.8 years, as well as 1,609,993 composite cardiovascular events. These figures appear difficult to reconcile with the stated patient population and follow-up duration. The authors should carefully recheck the calculations and clarify whether these are truly patient-level events/person-years or visit-level counts.**

**Response:**

We sincerely thank the reviewer for this careful and entirely correct observation. We rechecked all calculations and confirmed that the reported person-years and event numbers were erroneous.

The error arose during preparation of Table 3. Person-time was inadvertently computed by multiplying the visit-level denominator (21,000,013 M1 visits and 2,599,997 M2 visits) by the mean follow-up duration of 2.8 years, which produced the impossible figures of 58,800,036 and 7,279,991 “person-years”; the event numbers reported in the same table were then back-calculated from those inflated denominators. Because numerator and denominator were inflated by the same factor, the incidence rates per 1000 person-years were not affected, but the absolute counts and the person-time were reported at the wrong level of analysis. These were therefore visit-scaled, not patient-level, quantities, exactly as the reviewer suspected.

We have recalculated all time-to-event quantities strictly at the patient level. For each fee period, every patient contributing at least one outpatient visit in that period was followed from the first qualifying visit until the earliest occurrence of the event of interest, loss of database observation, or end of the period. This yields 551,000 patients and 1,542,800 patient-years for M1 (mean follow-up 2.8 years) and 117,000 patients and 58,500 patient-years for M2 (mean follow-up 0.5 years, maximum 7 months). The corresponding composite cardiovascular event counts are 42,242 in M1 (7.7% of M1 patients) and 1591 in M2 (1.4% of M2 patients), giving 27.38 (95% CI 27.12–27.64) and 27.20 (95% CI 25.88–28.57) events per 1000 patient-years. All other outcomes were recalculated in the same way, and 95% CIs are now exact Poisson intervals rather than the previously reported approximate intervals, which were also inconsistent with the event counts.

Changes made: Table 3 has been reconstructed using patient-level numerators, patient-years, and exact Poisson CIs; the row label “Total person-years” is now “Total patient-years” and the column headings now read “No. of patients with event” and “Incidence rate per 1000 patient-years.” Footnote b now explicitly states the patient-level denominators. The Methods (Statistical analysis) now describe the patient-level follow-up algorithm, and corresponding sentences in the Results and Discussion, as well as in the Abstract, have been revised accordingly.

**2.Patient-level versus visit-level analysis remains problematic.
Although the manuscript now explicitly states that outpatient visits are the unit of observation, Table 1 and several sections still use “patients” when reporting millions of observations. For example, the reported 21,000,013 M1 and 2,599,997 M2 observations are visits, not patients. This terminology should be corrected consistently throughout the manuscript.**

**Response:**

We fully agree, and we apologize for the inconsistent terminology. We have systematically separated visit-level from patient-level reporting throughout the manuscript.

Specifically: (i) in Table 1, the row previously labelled “Total patients, n” now reads “Total outpatient visits, n” (21,000,013 and 2,599,997), and a new row, “Unique patients contributing visits, n” has been added (551,000 for M1 and 117,000 for M2); (ii) the Table 1 title now specifies “unit of observation: outpatient visits” and the footnote states that visit counts do not represent counts of distinct patients; (iii) the Table 2 column headings now state the number of outpatient visits in each fee period; (iv) Table 3 is now explicitly patient-level, as described in our response to point 1; (v) in the Results, we now state that 551,000 unique patients (472,000 with M1 visits only plus 79,000 with visits in both periods) contributed the 21,000,013 M1 visits and that 117,000 unique patients (38,000 with M2 visits only plus 79,000 with visits in both periods) contributed the 2,599,997 M2 visits; (vi) the Abstract, Discussion, and the Fig 1 legend now consistently refer to “outpatient visits” for all visit-level proportions and to “patients” only for patient-level quantities; and (vii) the Methods now include the explicit statement that “visit-level counts are reported as the number of outpatient visits and are never interpreted as the number of patients.”

We have also revised Fig 1 for the same reason. The figure now presents LDL-C goal attainment as the percentage of outpatient visits in each age stratum, the in-bar annotations are explicitly labeled as visits rather than sample sizes, and the legend states the number of unique patients contributing those visits. Additionally, because repeated visits are clustered within patients, the 95% CIs shown in Fig 1 are now calculated using the number of unique patients contributing visits in each stratum as the effective sample size, which is a deliberately conservative choice; the previous error bars were not consistent with the number of observations. The figure has been regenerated as a 300-dpi RGB TIFF at 19.05 cm width, in accordance with the *PLOS ONE* figure guidelines.

**3.The abstract contains an apparent numerical inconsistency.
The abstract states that approximately 50% of patients achieved the LDL-C goal, whereas the Results report 58.3% and 58.5% of M1 and M2 visits, respectively. The authors should reconcile these numbers and clearly specify whether the denominator is patients or visits.**

**Response:**

Thank you for catching this. The statement that “approximately 50% of patients achieved their low-density lipoprotein cholesterol goal” was an outdated sentence retained from an earlier draft and was both numerically wrong and expressed with the wrong denominator.

The Abstract now reads: “Low-density lipoprotein cholesterol goals were attained at 58.3% of outpatient visits under the previous fee and 58.5% under the new fee,” which matches the Results and Table 2 exactly and specifies visits as the denominator. The Abstract also now explicitly states the cohort structure (590,000 patients contributing 23,600,010 outpatient visits) and specifies that visits were the unit of observation for lipid and fee-category analyses whereas cardiovascular outcomes and patient-time were analyzed at the patient level. We have rechecked every value reported in the Abstract against the Results and tables; the revised Abstract is 300 words in length and therefore remains within the *PLOS ONE* limit.

**4.The comparison between M1 and M2 should be interpreted more cautiously.
M1 covers January 2021–May 2024, whereas M2 covers only June–December 2024. Moreover, patients could contribute observations to both periods. Therefore, these are not independent groups, and simple cross-sectional comparisons may be affected by repeated observations and temporal differences. The limitations of this approach should be emphasized more explicitly.**

**Response:**

We agree entirely and have emphasized this caution rather than merely acknowledging it.

In the Methods, we now state that, because a patient could contribute visits to both fee periods and multiple visits within the same period, M1 and M2 are not independent samples and visit-level proportions are clustered within patients; for this reason, we report conservative 95% CIs based on the number of unique patients contributing visits and we deliberately refrained from formal hypothesis testing of policy effects.

In the Limitations, we have added a paragraph explicitly quantifying the problem: M1 spans January 2021 to May 2024 (41 months) whereas M2 only covers June to December 2024 (7 months), and 79,000 patients (13% of the cohort) contributed observations to both periods. We now state that the M1 versus M2 contrasts are repeated cross-sectional descriptions of the same evolving population rather than comparisons of independent groups, and that these remain vulnerable to secular trends, calendar-time and seasonal effects in lipid testing, and within-patient correlation of repeated visits. All M1 versus M2 differences are now framed as descriptive throughout the manuscript.

**5.The M2 cardiovascular outcome analysis is particularly limited.
Cardiovascular outcomes after implementation of M2 are available for only approximately six months. Consequently, comparison of cardiovascular event rates between M1 and M2 may be highly susceptible to differences in follow-up duration and should not be interpreted as evidence that the new fee structure had no effect on cardiovascular outcomes.**

**Response:**

We agree, and we have removed any wording that could be read as evidence of no effect.

The revised Results now state that only 58,500 patient-years accumulated in M2 versus 1,542,800 in M1, approximately 26-fold fewer patient-years, and that “this similarity should be regarded as descriptive only and must not be read as evidence that the fee revision had no effect on cardiovascular outcomes.” The Discussion now states that this number of patient-years is insufficient to detect a change in event rates and that “the absence of an observed difference is uninformative rather than reassuring.” Footnote b of Table 3 states the same caveat, and this is repeated in a new paragraph in the Limitations. Appropriately modified text has been added to the Abstract, which now notes that the outcome comparison is exploratory and cannot exclude an effect of the new fee structure. The concluding statements of the manuscript now refer to LDL-C control rather than to cardiovascular outcomes.

**6. Claims-data limitations should be expanded.
The MDV database excludes most small clinics and non-DPC facilities, which may limit generalizability to the overall Japanese dyslipidemia population. In addition, lifestyle counseling, adherence, dietary modification, physical activity, and other components of the new management strategy are not directly measurable in claims data.**

**Response:**

Thank you for this suggestion, which we have adopted in full.

The Limitations now state that the MDV database is built on DPC-participating acute care hospitals and therefore excludes most small clinics, primary care practices, and other non-DPC facilities, where a large share of routine dyslipidemia care and much of the uptake of the lifestyle disease management fee is likely to occur; that the cohort is consequently enriched for older, multimorbid, hospital-managed patients; and that the findings may not generalize to the overall Japanese population with dyslipidemia.

We further state that the central components of the new management strategy—structured lifestyle counseling, shared goal setting recorded on the Treatment Plan Sheet, medication adherence, dietary modification, physical activity, sleep, smoking behavior, and body weight—are not directly measurable using administrative claims; that adherence could only be approximated from prescription continuity and the delivery or quality of counseling could not be observed at all; that laboratory values are recorded only for tests performed at participating institutions and may therefore not be missing at random; and that coding of the fee category during the early implementation phase may be subject to misclassification.

**7. To improve all section add "ÇOLAK, Ayşe, et al. Efficacy and safety of combination therapy with statin and ezetimibe in patients failing to achieve target LDL levels at cardiology outpatient clinics in Turkey (COM-TR-OLDL): a real-world observational study. Lipids in Health and Disease, 2026."
Overall recommendation.
The study addresses an important and timely question and has substantial potential because of the size of the database. However, the apparent inconsistencies in the cardiovascular event/person-year calculations and the persistent ambiguity between patient-level and visit-level denominators are important enough to require correction before the manuscript can be considered further.**

**Response:**

We thank the reviewer for directing us to this highly relevant study, which we have read and now cite.

The reference has been added as reference 23: Çolak A, Kumral Z, Öztürk Ö, Ülgen Kunak A, Elmas AN, Bedir Ö, et al. Efficacy and safety of combination therapy with statin and ezetimibe in patients failing to achieve target LDL levels at cardiology outpatient clinics in Turkey (COM-TR-OLDL): a real-world observational study. Lipids Health Dis. 2026;25(1):159. https://doi.org/10.1186/s12944-026-02950-1

This reference is cited in the Introduction, where we note that recent real-world data from cardiology outpatient clinics in Turkey similarly showed that many patients remain above target despite intensified statin-based therapy and that adding ezetimibe improved but did not normalize goal attainment; this work is referenced again in the Discussion, where we note that our findings are consistent with contemporary real-world evidence from other health systems in which a large share of patients attending specialist outpatient clinics remained above target despite intensification of statin-based regimens. This study is particularly apposite to our results because ezetimibe was used at 27.6%–27.7% of visits in our cohort, yet LDL-C goals were still unmet at more than 40% of visits.

We are grateful for this constructive assessment. The two issues identified as decisive—the cardiovascular event and person-year calculations, and the ambiguity between patient-level and visit-level denominators—have both been corrected at the source rather than merely qualified in the text. Person-time and event counts are now derived strictly at the patient level (551,000 patients and 1,542,800 patient-years for M1; 117,000 patients and 58,500 patient-years for M2); every visit-level figure is now labeled as outpatient visits; and the number of unique patients underlying each visit count is reported. The Abstract has been reconciled with the Results; the M1 versus M2 comparison is now framed as a non-independent, unequal-length descriptive contrast; the cardiovascular comparison is explicitly stated to be uninformative rather than null; the claims-data limitations have been expanded; and the suggested reference has been incorporated. Figure 1 has also been corrected and recreated to meet PLOS ONE specifications. We hope the revised manuscript is now suitable for publication, and we thank the reviewer for the rigor that has led to these improvements.

**7. PLOS authors have the option to publish the peer review history of their article (**[**what does this mean?**](https://track.editorialmanager.com/CL0/https:%2F%2Fjournals.plos.org%2Fplosone%2Fs%2Feditorial-and-peer-review-process%23loc-peer-review-history/1/010f01a03686b21b-a89338d0-4e82-4e4d-ad00-2a28b0d445d2-000000/rsE5XNPGX98MyDjO2nByGRQeQTdS4fvxIaSwMaxcVIw=258)**). If published, this will include your full peer review and any attached files.**

**If you choose “no”, your identity will remain anonymous but your review may still be made public.

Do you want your identity to be public for this peer review? For information about this choice, including consent withdrawal, please see our** [**Privacy Policy**](https://track.editorialmanager.com/CL0/https:%2F%2Fwww.plos.org%2Fprivacy-policy/1/010f01a03686b21b-a89338d0-4e82-4e4d-ad00-2a28b0d445d2-000000/sdQfJOMqwqpaAEfV7rS6G18Fq7B4GdAHmBuGfSrFSdw=258)**.**

**Reviewer #1: Yes: DR FARHANA FAKHIRA ISMAIL**

**Reviewer #3: No**
