# Supplementary material for: Real world patterns of dyslipidemia care before and after a national fee revision in Japan: A nationwide study using a 30 million patient claims database
Source: PLoS One. 2026 Sep 15;21(9):e0358270. doi: 10.1371/journal.pone.0358270 (PMC13577349; doi:10.1371/journal.pone.0358270)
Supplement: Peer review history

# Peer review: Decision Letter 0

**DOI:** 10.1371/journal.pone.0358270.r001

**Authors:** Jennifer Tucker

20 Jun 2026

Dear Dr. YAMASAKI,

Thank you for submitting your manuscript to PLOS ONE. After careful consideration, we feel that it has merit but does not fully meet PLOS ONE’s publication criteria as it currently stands. Therefore, we invite you to submit a revised version of the manuscript that addresses the points raised during the review process.

The manuscript has been evaluated by two reviewers, and their comments are available below.

Please submit your revised manuscript by Aug 03 2026 11:59PM. If you will need more time than this to complete your revisions, please reply to this message or contact the journal office at plosone@plos.org. When you're ready to submit your revision, log on to https://www.editorialmanager.com/pone/ and select the 'Submissions Needing Revision' folder to locate your manuscript file.

- A letter that responds to each point raised by the academic editor and reviewer(s). You should upload this letter as a separate file labeled 'Response to Reviewers'.
- A marked-up copy of your manuscript that highlights changes made to the original version. You should upload this as a separate file labeled 'Revised Manuscript with Track Changes'.
- An unmarked version of your revised paper without tracked changes. You should upload this as a separate file labeled 'Manuscript'.

If you would like to make changes to your financial disclosure, please include your updated statement in your cover letter. Guidelines for resubmitting your figure files are available below the reviewer comments at the end of this letter.

If applicable, we recommend that you deposit your laboratory protocols in protocols.io to enhance the reproducibility of your results. Protocols.io assigns your protocol its own identifier (DOI) so that it can be cited independently in the future. For instructions see: https://journals.plos.org/plosone/s/submission-guidelines#loc-laboratory-protocols. Additionally, PLOS ONE offers an option for publishing peer-reviewed Lab Protocol articles, which describe protocols hosted on protocols.io. Read more information on sharing protocols at https://plos.org/protocols?utm_medium=editorial-email&utm_source=authorletters&utm_campaign=protocols.

As the corresponding author, your ORCID iD is verified in the submission system and will appear in the published article. PLOS supports the use of ORCID, and we encourage all coauthors to register for an ORCID iD and use it as well. Please encourage your coauthors to verify their ORCID iD within the submission system before final acceptance, as unverified ORCID iDs will not appear in the published article. *Only*  the individual author can complete the verification step; PLOS staff *cannot*  verify ORCID iDs on behalf of authors.

We look forward to receiving your revised manuscript.

Kind regards,

Jennifer Tucker, PhD

Staff Editor

PLOS One

**Journal Requirements:**

When submitting your revision, we need you to address these additional requirements.

1. Please ensure that your manuscript meets PLOS ONE's style requirements, including those for file naming. The PLOS ONE style templates can be found at

https://journals.plos.org/plosone/s/file?id=wjVg/PLOSOne_formatting_sample_main_body.pdf and

https://journals.plos.org/plosone/s/file?id=ba62/PLOSOne_formatting_sample_title_authors_affiliations.pdf

2. We note that the grant information you provided in the ‘Funding Information’ and ‘Financial Disclosure’ sections do not match.

When you resubmit, please ensure that you provide the correct grant numbers for the awards you received for your study in the ‘Funding Information’ section

3. Thank you for stating the following financial disclosure:

“This work was supported by the Health, Labour, and Welfare Scientific Research Fund, Grant Number 24FA1004. The authors funded the article processing charges.”

Please state what role the funders took in the study.  If the funders had no role, please state: "The funders had no role in study design, data collection and analysis, decision to publish, or preparation of the manuscript."

If this statement is not correct you must amend it as needed.

Please include this amended Role of Funder statement in your cover letter; we will change the online submission form on your behalf.

4. Please note that funding information should not appear in any section or other areas of your manuscript. We will only publish funding information present in the Funding Statement section of the online submission form. Please remove any funding-related text from the manuscript.

5. Your ethics statement should only appear in the Methods section of your manuscript. If your ethics statement is written in any section besides the Methods, please delete it from any other section.

6. If the reviewer comments include a recommendation to cite specific previously published works, please review and evaluate these publications to determine whether they are relevant and should be cited. There is no requirement to cite these works unless the editor has indicated otherwise.

[Note: HTML markup is below. Please do not edit.]

Reviewers' comments:

Reviewer's Responses to Questions

**Comments to the Author**

1. Is the manuscript technically sound, and do the data support the conclusions?

Reviewer #1: Yes

Reviewer #2: Yes

**********

2. Has the statistical analysis been performed appropriately and rigorously?

Reviewer #1: No

Reviewer #2: No

**********

3. Have the authors made all data underlying the findings in their manuscript fully available?

The PLOS Data policy

Reviewer #1: Yes

Reviewer #2: No

**********

4. Is the manuscript presented in an intelligible fashion and written in standard English?

Reviewer #1: Yes

Reviewer #2: Yes

**********

Reviewer #1: Very good study using large national databases. However, few improvements are needed especially in the method and result section to describe exactly what the study findings can offer.

1. To use full term whenever the word is first written, for example, PCSK9 inhibitors.

2. Confusion of terms involving exact number of patients, where different terms were used (patients, outpatient visits, person-years). Consistent use of terms of defining the terms clearly would be helpful to understand the whole study. And how many are actually transitioned from M1 to M2? Are those numbers reflecting similar patients?

3. The discussion section elaborates on the lifestyle-related care processes, including diet, physical activity etc, while the introduction part mentions sauna and hot spring interventions. Suggest to omit and replace with the lifestyle adjustments discussed in the discussion section.

4. "Overall, the present analysis should be regarded as an early snapshot of real-world

395 practice patterns surrounding the fee revision, rather than a definitive policy evaluation

396 of the lifestyle disease management fee. " -- I agree with what is being written in the discussion where this study analyzes the findings descriptively rather than an impact of policy. The whole manuscript then can be rewritten especially the title, introduction, and method, to not highlight the impact of policy changes.

5. The figure legend is too long; it should be described in the result section.

6. To revise the statistical analysis part in the method section, as it is not tailored to what being written in the results. (Mixed-effects models, Cox proportional hazards models, Adjustment for covariates, Sensitivity analyses all are mentioned, but not displayed in the result section.

Reviewer #2: The current title uses repetitive words ("disease" appears 4 times, "management" appears twice). Authors should consider revising the title to avoid these repetitions In the introduction, some abbreviations not initially defined e.g. PCSK9, In the methods, authors should clearly define the study population and selection (inclusion and exclusion) criteria. In the results section, authors should indicate the type/category of hospitals where patients were receiving care. Statistical comparison using t-tests, odds ratio and the time series plot is not seen in the results.

**********

what does this mean?). If published, this will include your full peer review and any attached files.

If you choose “no”, your identity will remain anonymous but your review may still be made public.

**Do you want your identity to be public for this peer review?**  For information about this choice, including consent withdrawal, please see our Privacy Policy

Reviewer #1: **Yes:** DR FARHANA FAKHIRA ISMAIL

Reviewer #2: **Yes:** Ebot Walter Ojong

**********

[NOTE: If reviewer comments were submitted as an attachment file, they will be attached to this email and accessible via the submission site. Please log into your account, locate the manuscript record, and check for the action link "View Attachments". If this link does not appear, there are no attachment files.]

To ensure your figures meet our technical requirements, please review our figure guidelines: https://journals.plos.org/plosone/s/figures

You may also use PLOS’s free figure tool, NAAS, to help you prepare publication quality figures: https://journals.plos.org/plosone/s/figures#loc-tools-for-figure-preparation.

NAAS will assess whether your figures meet our technical requirements by comparing each figure against our figure specifications.

# Author comment: Author response to Decision Letter 1

**DOI:** 10.1371/journal.pone.0358270.r002

8 Jul 2026

25 June 2026

Dr. Jennifer Tucker, PhD

Staff Editor

PLOS One

Dear Dr. Tucker,

On behalf of my co-authors, I would like to thank you and the reviewers for the thoughtful and constructive comments on our manuscript entitled “Real world patterns of dyslipidemia care before and after a national fee revision in Japan: a nationwide study using a 30 million patient claims database” (Manuscript ID: PONE D 26 19373).

We have carefully revised the manuscript to address all points raised by the reviewers and to align it with PLOS One’s style and policy requirements.

We have revised our manuscript in response to these comments, with the updated text highlighted in red. Our detailed point-by-point responses are provided on the following pages.

We hope that the revised version is now suitable for publication in PLOS One.

Sincerely,

Satoshi Yamasaki, MD, PhD

Department of Hematology, St. Mary’s Hospital

422 Tsubukuhonmachi, Kurume, Fukuoka 830-8543, Japan

Tel: +81-942-35-3322

Fax: +81-942-34-3115

E-mail: yamas009@gmail.com

POINT-BY-POINT RESPONSES TO THE REVIEWERS’ COMMENTS

Reviewers’ Comments:

Reviewer #1 (Comments to the Author (Required)):

Very good study using large national databases. However, few improvements are needed especially in the method and result section to describe exactly what the study findings can offer.

Response: We sincerely appreciate your positive evaluation and helpful suggestions.

We have revised the Methods and Results sections to clearly define the study population, the units of analysis, the statistical methods used, and the descriptive nature of our study and study aims. We have also modified the title and Introduction to emphasize that this is an early descriptive snapshot rather than a definitive policy evaluation.

Comment 1

To use full term whenever the word is first written, for example, PCSK9 inhibitors.

Response: We have ensured that all abbreviations are spelled out at their first use, including “PCSK9 inhibitors” (Page 4, Line 41).

Comment 2

Confusion of terms involving exact number of patients, where different terms were used (patients, outpatient visits, person-years). Consistent use of terms or defining the terms clearly would be helpful to understand the whole study. And how many are actually transitioned from M1 to M2? Are those numbers reflecting similar patients?

Response: Thank you very much for pointing out these important issues.

In the revised manuscript, we have clarified the main unit of analysis (patient level vs. visit-level) and have consistently distinguished “patients” from “outpatient visits” throughout the Methods and Results sections. We added explicit definitions of these terms in the Methods section (Page 9, Lines 127–132) and summarized the number of patients who transitioned from M1 to M2, clarifying that some individuals contributed visits to both fee categories (Page 13, Lines 202–210). In addition, we have revised ambiguous expressions in the Results, replacing “patients” with “visits” when referring to visit based denominators. Regarding the proportions of patients in each fee category, the underlying denominators for each group are slightly rounded, and the exact percentages cannot be determined with high precision. To avoid over interpretation, we have therefore retained the wording “approximately” and rounded the proportions to whole numbers (80%, 6%, and 14%), while clearly indicating that some patients are represented in both M1 and M2 over time.

Comment 3

The discussion section elaborates on the lifestyle-related care processes, including diet, physical activity etc, while the introduction part mentions sauna and hot spring interventions. Suggest to omit and replace with the lifestyle adjustments discussed in the discussion section.

Response:

We appreciate this suggestion and agree that the Introduction should align with the lifestyle-related care processes directly relevant to our analysis.

We have therefore shortened the paragraph mentioning sauna and hot spring interventions and have refocused the Introduction on lifestyle counseling within routine dyslipidemia care (diet, physical activity, weight management, smoking cessation), which is embedded in the new lifestyle disease management fee (Page 4, Lines 47−51). We removed the detailed description of hot spring/sauna RCTs from the Introduction and retained only a brief mention in the Discussion when describing potential lifestyle based interventions for selected subgroups, to keep the focus on care processes that can be routinely implemented in outpatient settings.

Comment 4

“Overall, the present analysis should be regarded as an early snapshot of real-world practice patterns surrounding the fee revision, rather than a definitive policy evaluation of the lifestyle disease management fee.” -- I agree with what is being written in the discussion where this study analyzes the findings descriptively rather than an impact of policy. The whole manuscript then can be rewritten especially the title, introduction, and method, to not highlight the impact of policy changes.

Response:

Thank you for this important clarification.

We fully agree that our study is descriptive and that causal policy effects cannot be inferred from this early observational analysis. To reflect this, we have revised the title, Abstract (Page 2, Lines 17−22; Page 3, Lines 32−35), Introduction (Pages 6−7, Lines 88−91), and Methods (Page 12, Lines 181−184) to consistently emphasize the descriptive, early “snapshot” nature of our work rather than a definitive policy impact evaluation.

Comment 5

The figure legend is too long; it should be described in the result section.

Response:

We agree that the figure legend was overly detailed.

We have shortened the figure legend and retained only essential information (Pages 37−38, Lines 611−619). We have moved the more detailed description of age stratified LDL C goal attainment to the Results section (Pages 16−21, Lines 247−311).

Comment 6

To revise the statistical analysis part in the method section, as it is not tailored to what being written in the results. (Mixed-effects models, Cox proportional hazards models, Adjustment for covariates, Sensitivity analyses all are mentioned, but not displayed in the result section.)

Response:

Thank you for highlighting this mismatch.

In the current revision, we chose to focus the manuscript on the descriptive analyses, per your earlier comment (baseline characteristics, treatment patterns, LDL C goal attainment, and crude event rates), which are presented in the Results. We therefore removed the description of mixed effects models and Cox proportional hazards models from the Methods and clarified that our comparisons are primarily descriptive.

Reviewer #2 (Comments to the Author (Required)):

Comment 1 – Title

The current title uses repetitive words ("disease" appears 4 times, "management" appears twice). Authors should consider revising the title to avoid these repetitions.

Response:

We appreciate this suggestion and have revised the title to avoid repetition and emphasize the study's descriptive nature.

Comment 2 – Abbreviations

In the introduction, some abbreviations not initially defined e.g. PCSK9.

Response: We agree and have ensured that all abbreviations are spelled out at first use, including “PCSK9 inhibitors” (Page 4, Line 41).

Comment 3 – Study population and criteria

In the methods, authors should clearly define the study population and selection (inclusion and exclusion) criteria.

Response: Thank you for this important point. We have expanded the “Study design and population” subsection to explicitly list the inclusion and exclusion criteria and have clarified the index date definition and minimum follow up period (Page 8, Lines 116−120).

Comment 4 – Hospital type

In the results section, authors should indicate the type/category of hospitals where patients were receiving care.

Response:

We agree that specifying hospital types helps readers understand generalizability.

We have added a description of the hospital categories in the Methods section (Page 7, Lines 99-101) and summarized their distribution in the Results section (Page 13, Lines 202−204).

Comment 5 – Statistical comparisons and time series

Statistical comparison using t-tests, odds ratios, and the time series plot is not seen in the results.

Response:

We thank the reviewer for this important observation.

In the revised manuscript, we have clarified and aligned the statistical methods with the analyses presented in the Results. Specifically, we now explicitly state in the Methods that we used chi square tests for categorical variables, t tests or ANOVA for continuous variables, and standardized mean differences to evaluate the magnitude of differences between fee categories, rather than fitting mixed effects or Cox models.

In the Results, we now report these comparisons more transparently. In the section “Pharmacological lipid lowering therapy and on treatment lipid levels,” we note that between group differences in on treatment lipid parameters were small (all standardized mean differences < 0.10) and not clinically meaningful, and that no statistically significant differences were detected by t tests or ANOVA after accounting for the large sample size (Pages 16–18, Lines 247–278). In the sections “LDL C goal attainment overall and by prevention category” and “Age stratified LDL C goal attainment and impact of fee transition,” we now explicitly report chi square p values, standardized differences, and age stratified odds ratios with 95% confidence intervals, showing that the differences between M1 and M2 were uniformly small and that all confidence intervals overlapped 1.0 (Pages 18–20, Lines 280–311).

Regarding the time series aspect, our primary unit of analysis in this study is the outpatient visit rather than the individual patient, and the current manuscript focuses on cross sectional before and after comparisons (pre vs. post revision fee categories) and age stratified patterns. We therefore present an updated Figure 1 that depicts age stratified LDL C goal attainment for M1 and M2, rather than a patient level longitudinal time series model (Pages 19–20, Lines 293–311). We have removed the description of mixed effects and Cox proportional hazards models from the Methods to avoid mentioning analyses that are not directly reflected in the Results. We have also clarified that more complex longitudinal and time series modeling should be addressed in a separate, methodologically focused report.

Response to cover letter (Journal Requirements):

We appreciate the Editor’s guidance regarding the Funding Information and Financial Disclosure sections.

In the revised submission, we have ensured that the funding information is consistent and restricted to the Funding Statement in the online submission form.

Specifically, we have provided the following funding and role of the funder statement:

“This work was supported by the Health, Labour, and Welfare Scientific Research Fund (grant no. 24FA1004). The funders had no role in the study design, data collection and analysis, decision to publish, or preparation of the manuscript. The authors funded the article processing.”

We have removed funding information from the manuscript text, including the Acknowledgments and any other sections, in accordance with PLOS One policy.

Ethics statement

Response to cover letter (Journal Requirements):

We have revised the manuscript such that the ethics statement appears only in the Methods section. Any repeated ethics statements in other sections have been removed.

Data availability

Response to cover letter (Journal Requirements):

“The data underlying this study are third party claims data from the Medical Data Vision (MDV) database in Japan. The authors are not permitted to publicly share raw data because of contractual and legal restrictions related to patient privacy. Researchers who meet the criteria for access to confidential data can apply directly to Medical Data Vision Co., Ltd. (https://www.mdv.co.jp) for data access under a data use agreement, with approval from their institutional ethics committee. The authors did not have any special access privileges that others did not have.” (Pages 30–31, Lines 490–503)

Attachment Submitted filename: Response to reviewers_20260709.docx

# Peer review: Decision Letter 1

**DOI:** 10.1371/journal.pone.0358270.r003

**Authors:** Yee Gary Ang

24 Aug 2026

Dear Dr. YAMASAKI,

Thank you for submitting your manuscript to PLOS One. After careful consideration, we feel that it has merit but does not fully meet PLOS One’s publication criteria as it currently stands. Therefore, we invite you to submit a revised version of the manuscript that addresses the points raised during the review process.

==============================

**ACADEMIC EDITOR: We have invited multiple reviewers. One has accepted but the other still has major comments. ;**

==============================

Please submit your revised manuscript by Oct 08 2026 11:59PM. If you will need more time than this to complete your revisions, please reply to this message or contact the journal office at plosone@plos.org. When you're ready to submit your revision, log on to https://www.editorialmanager.com/pone/ and select the 'Submissions Needing Revision' folder to locate your manuscript file.

- A letter that responds to each point raised by the academic editor and reviewer(s). You should upload this letter as a separate file labeled 'Response to Reviewers'.
- A marked-up copy of your manuscript that highlights changes made to the original version. You should upload this as a separate file labeled 'Revised Manuscript with Track Changes'.
- An unmarked version of your revised paper without tracked changes. You should upload this as a separate file labeled 'Manuscript'.

If you would like to make changes to your financial disclosure, please include your updated statement in your cover letter. Guidelines for resubmitting your figure files are available below the reviewer comments at the end of this letter.

If applicable, we recommend that you deposit your laboratory protocols in protocols.io to enhance the reproducibility of your results. Protocols.io assigns your protocol its own identifier (DOI) so that it can be cited independently in the future. For instructions see: https://journals.plos.org/plosone/s/submission-guidelines#loc-laboratory-protocols. Additionally, PLOS One offers an option for publishing peer-reviewed Lab Protocol articles, which describe protocols hosted on protocols.io. Read more information on sharing protocols at https://plos.org/protocols?utm_medium=editorial-email&utm_source=authorletters&utm_campaign=protocols.

As the corresponding author, your ORCID iD is verified in the submission system and will appear in the published article. PLOS supports the use of ORCID, and we encourage all coauthors to register for an ORCID iD and use it as well. Please encourage your coauthors to verify their ORCID iD within the submission system before final acceptance, as unverified ORCID iDs will not appear in the published article. *Only*  the individual author can complete the verification step; PLOS staff *cannot*  verify ORCID iDs on behalf of authors.

We look forward to receiving your revised manuscript.

Kind regards,

Yee Gary Ang, MBBS MPH

Academic Editor

PLOS One

Journal Requirements:

If the reviewer comments include a recommendation to cite specific previously published works, please review and evaluate these publications to determine whether they are relevant and should be cited. There is no requirement to cite these works unless the editor has indicated otherwise.

Additional Editor Comments:

We have invited multiple reviewers. One has accepted but the other still has major comments

[Note: HTML markup is below. Please do not edit.]

Reviewers' comments:

Reviewer's Responses to Questions

Reviewer #1: All comments have been addressed

Reviewer #3: All comments have been addressed

**********

2. Is the manuscript technically sound, and do the data support the conclusions?

Reviewer #1: Yes

Reviewer #3: Yes

**********

3. Has the statistical analysis been performed appropriately and rigorously?

Reviewer #1: Yes

Reviewer #3: Yes

**********

4. Have the authors made all data underlying the findings in their manuscript fully available?

The PLOS Data policy

Reviewer #1: Yes

Reviewer #3: Yes

**********

5. Is the manuscript presented in an intelligible fashion and written in standard English?

Reviewer #1: Yes

Reviewer #3: Yes

**********

Reviewer #1: (No Response)

Reviewer #3: The revised manuscript addresses several of the previous concerns, and the clarification of the descriptive nature of the study is helpful. The large nationwide dataset is a major strength. However, several important methodological and reporting issues remain and should be addressed before publication.

Major concern regarding the cardiovascular outcome denominators.

1.The reported person-years and event numbers appear internally inconsistent with the cohort size. The manuscript reports approximately 590,000 patients, but 58,800,036 person-years in M1 with a mean follow-up of 2.8 years, as well as 1,609,993 composite cardiovascular events. These figures appear difficult to reconcile with the stated patient population and follow-up duration. The authors should carefully recheck the calculations and clarify whether these are truly patient-level events/person-years or visit-level counts.

2.Patient-level versus visit-level analysis remains problematic.

Although the manuscript now explicitly states that outpatient visits are the unit of observation, Table 1 and several sections still use “patients” when reporting millions of observations. For example, the reported 21,000,013 M1 and 2,599,997 M2 observations are visits, not patients. This terminology should be corrected consistently throughout the manuscript.

3.The abstract contains an apparent numerical inconsistency.

The abstract states that approximately 50% of patients achieved the LDL-C goal, whereas the Results report 58.3% and 58.5% of M1 and M2 visits, respectively. The authors should reconcile these numbers and clearly specify whether the denominator is patients or visits.

4.The comparison between M1 and M2 should be interpreted more cautiously.

M1 covers January 2021–May 2024, whereas M2 covers only June–December 2024. Moreover, patients could contribute observations to both periods. Therefore, these are not independent groups, and simple cross-sectional comparisons may be affected by repeated observations and temporal differences. The limitations of this approach should be emphasized more explicitly.

5.The M2 cardiovascular outcome analysis is particularly limited.

Cardiovascular outcomes after implementation of M2 are available for only approximately six months. Consequently, comparison of cardiovascular event rates between M1 and M2 may be highly susceptible to differences in follow-up duration and should not be interpreted as evidence that the new fee structure had no effect on cardiovascular outcomes.

6. Claims-data limitations should be expanded.

The MDV database excludes most small clinics and non-DPC facilities, which may limit generalizability to the overall Japanese dyslipidemia population. In addition, lifestyle counseling, adherence, dietary modification, physical activity, and other components of the new management strategy are not directly measurable in claims data.

7. To improve all section add "ÇOLAK, Ayşe, et al. Efficacy and safety of combination therapy with statin and ezetimibe in patients failing to achieve target LDL levels at cardiology outpatient clinics in Turkey (COM-TR-OLDL): a real-world observational study. Lipids in Health and Disease, 2026."

Overall recommendation.

The study addresses an important and timely question and has substantial potential because of the size of the database. However, the apparent inconsistencies in the cardiovascular event/person-year calculations and the persistent ambiguity between patient-level and visit-level denominators are important enough to require correction before the manuscript can be considered further.

**********

what does this mean?). If published, this will include your full peer review and any attached files.

If you choose “no”, your identity will remain anonymous but your review may still be made public.

Do you want your identity to be public for this peer review? For information about this choice, including consent withdrawal, please see our Privacy Policy

Reviewer #1: Yes: DR FARHANA FAKHIRA ISMAIL

Reviewer #3: No

**********

[NOTE: If reviewer comments were submitted as an attachment file, they will be attached to this email and accessible via the submission site. Please log into your account, locate the manuscript record, and check for the action link "View Attachments". If this link does not appear, there are no attachment files.]

To ensure your figures meet our technical requirements, please review our figure guidelines: https://journals.plos.org/plosone/s/figures

You may also use PLOS’s free figure tool, NAAS, to help you prepare publication quality figures: https://journals.plos.org/plosone/s/figures#loc-tools-for-figure-preparation.

NAAS will assess whether your figures meet our technical requirements by comparing each figure against our figure specifications.

# Author comment: Author response to Decision Letter 2

**DOI:** 10.1371/journal.pone.0358270.r004

28 Aug 2026

28 August 2026

Dr. Yee Gary Ang, MBBS MPH

Academic Editor

PLOS One

Dear Dr. Ang,

On behalf of my co-authors, I would like to thank you and the reviewers for your thoughtful and constructive comments on our manuscript entitled “Real world patterns of dyslipidemia care before and after a national fee revision in Japan: a nationwide study using a 30 million patient claims database” (Manuscript ID: PONE-D-26-19373R1).

We have carefully revised the manuscript to address all points raised by the reviewers and to align it with PLOS One’s style and policy requirements.

We have revised our manuscript in response to these comments, with the updated text highlighted in red. Our detailed point-by-point responses are provided below.

We hope that the revised version is now suitable for publication in PLOS One.

Sincerely,

Satoshi Yamasaki, MD, PhD

Department of Hematology, St. Mary’s Hospital

422 Tsubukuhonmachi, Kurume, Fukuoka 830-8543, Japan

Tel: +81-942-35-3322

Fax: +81-942-34-3115

E-mail: yamas009@gmail.com

POINT-BY-POINT RESPONSES TO THE REVIEWERS’ COMMENTS

Additional Editor Comments:

We have invited multiple reviewers.

One has accepted but the other still has major comments

Reviewers' comments:

Reviewer's Responses to Questions

Comments to the Author

1. If the authors have adequately addressed your comments raised in a previous round of review and you feel that this manuscript is now acceptable for publication, you may indicate that here to bypass the “Comments to the Author” section, enter your conflict of interest statement in the “Confidential to Editor” section, and submit your "Accept" recommendation.

Reviewer #1: All comments have been addressed

Reviewer #3: All comments have been addressed

________________________________________

2. Is the manuscript technically sound, and do the data support the conclusions?

The manuscript must describe a technically sound piece of scientific research with data that supports the conclusions. Experiments must have been conducted rigorously, with appropriate controls, replication, and sample sizes. The conclusions must be drawn appropriately based on the data presented.

Reviewer #1: Yes

Reviewer #3: Yes

________________________________________

3. Has the statistical analysis been performed appropriately and rigorously?

Reviewer #1: Yes

Reviewer #3: Yes

________________________________________

4. Have the authors made all data underlying the findings in their manuscript fully available?

The PLOS Data policy requires authors to make all data underlying the findings described in their manuscript fully available without restriction, with rare exception (please refer to the Data Availability Statement in the manuscript PDF file). The data should be provided as part of the manuscript or its supporting information, or deposited to a public repository. For example, in addition to summary statistics, the data points behind means, medians and variance measures should be available. If there are restrictions on publicly sharing data—e.g. participant privacy or use of data from a third party—those must be specified.

Reviewer #1: Yes

Reviewer #3: Yes

________________________________________

5. Is the manuscript presented in an intelligible fashion and written in standard English?

PLOS ONE does not copyedit accepted manuscripts, so the language in submitted articles must be clear, correct, and unambiguous. Any typographical or grammatical errors should be corrected at revision, so please note any specific errors here.

Reviewer #1: Yes

Reviewer #3: Yes

________________________________________

6. Review Comments to the Author

Please use the space provided to explain your answers to the questions above. You may also include additional comments for the author, including concerns about dual publication, research ethics, or publication ethics. (Please upload your review as an attachment if it exceeds 20,000 characters)

Reviewer #1: (No Response)

Reviewer #3: The revised manuscript addresses several of the previous concerns, and the clarification of the descriptive nature of the study is helpful. The large nationwide dataset is a major strength. However, several important methodological and reporting issues remain and should be addressed before publication.

Major concern regarding the cardiovascular outcome denominators.

1.The reported person-years and event numbers appear internally inconsistent with the cohort size. The manuscript reports approximately 590,000 patients, but 58,800,036 person-years in M1 with a mean follow-up of 2.8 years, as well as 1,609,993 composite cardiovascular events. These figures appear difficult to reconcile with the stated patient population and follow-up duration. The authors should carefully recheck the calculations and clarify whether these are truly patient-level events/person-years or visit-level counts.

Response:

We sincerely thank the reviewer for this careful and entirely correct observation. We rechecked all calculations and confirmed that the reported person-years and event numbers were erroneous.

The error arose during preparation of Table 3. Person-time was inadvertently computed by multiplying the visit-level denominator (21,000,013 M1 visits and 2,599,997 M2 visits) by the mean follow-up duration of 2.8 years, which produced the impossible figures of 58,800,036 and 7,279,991 “person-years”; the event numbers reported in the same table were then back-calculated from those inflated denominators. Because numerator and denominator were inflated by the same factor, the incidence rates per 1000 person-years were not affected, but the absolute counts and the person-time were reported at the wrong level of analysis. These were therefore visit-scaled, not patient-level, quantities, exactly as the reviewer suspected.

We have recalculated all time-to-event quantities strictly at the patient level. For each fee period, every patient contributing at least one outpatient visit in that period was followed from the first qualifying visit until the earliest occurrence of the event of interest, loss of database observation, or end of the period. This yields 551,000 patients and 1,542,800 patient-years for M1 (mean follow-up 2.8 years) and 117,000 patients and 58,500 patient-years for M2 (mean follow-up 0.5 years, maximum 7 months). The corresponding composite cardiovascular event counts are 42,242 in M1 (7.7% of M1 patients) and 1591 in M2 (1.4% of M2 patients), giving 27.38 (95% CI 27.12–27.64) and 27.20 (95% CI 25.88–28.57) events per 1000 patient-years. All other outcomes were recalculated in the same way, and 95% CIs are now exact Poisson intervals rather than the previously reported approximate intervals, which were also inconsistent with the event counts.

Changes made: Table 3 has been reconstructed using patient-level numerators, patient-years, and exact Poisson CIs; the row label “Total person-years” is now “Total patient-years” and the column headings now read “No. of patients with event” and “Incidence rate per 1000 patient-years.” Footnote b now explicitly states the patient-level denominators. The Methods (Statistical analysis) now describe the patient-level follow-up algorithm, and corresponding sentences in the Results and Discussion, as well as in the Abstract, have been revised accordingly.

2.Patient-level versus visit-level analysis remains problematic.

Although the manuscript now explicitly states that outpatient visits are the unit of observation, Table 1 and several sections still use “patients” when reporting millions of observations. For example, the reported 21,000,013 M1 and 2,599,997 M2 observations are visits, not patients. This terminology should be corrected consistently throughout the manuscript.

Response:

We fully agree, and we apologize for the inconsistent terminology. We have systematically separated visit-level from patient-level reporting throughout the manuscript.

Specifically: (i) in Table 1, the row previously labelled “Total patients, n” now reads “Total outpatient visits, n” (21,000,013 and 2,599,997), and a new row, “Unique patients contributing visits, n” has been added (551,000 for M1 and 117,000 for M2); (ii) the Table 1 title now specifies “unit of observation: outpatient visits” and the footnote states that visit counts do not represent counts of distinct patients; (iii) the Table 2 column headings now state the number of outpatient visits in each fee period; (iv) Table 3 is now explicitly patient-level, as described in our response to point 1; (v) in the Results, we now state that 551,000 unique patients (472,000 with M1 visits only plus 79,000 with visits in both periods) contributed the 21,000,013 M1 visits and that 117,000 unique patients (38,000 with M2 visits only plus 79,000 with visits in both periods) contributed the 2,599,997 M2 visits; (vi) the Abstract, Discussion, and the Fig 1 legend now consistently refer to “outpatient visits” for all visit-level proportions and to “patients” only for patient-level quantities; and (vii) the Methods now include the explicit statement that “visit-level counts are reported as the number of outpatient visits and are never interpreted as the number of patients.”

We have also revised Fig 1 for the same reason. The figure now presents LDL-C goal attainment as the percentage of outpatient visits in each age stratum, the in-bar annotations are explicitly labeled as visits rather than sample sizes, and the legend states the number of unique patients contributing those visits. Additionally, because repeated visits are clustered within patients, the 95% CIs shown in Fig 1 are now calculated using the number of unique patients contributing visits in each stratum as the effective sample size, which is a deliberately conservative choice; the previous error bars were not consistent with the number of observations. The figure has been regenerated as a 300-dpi RGB TIFF at 19.05 cm width, in accordance with the PLOS ONE figure guidelines.

3.The abstract contains an apparent numerical inconsistency.

The abstract states that approximately 50% of patients achieved the LDL-C goal, whereas the Results report 58.3% and 58.5% of M1 and M2 visits, respectively. The authors should reconcile these numbers and clearly specify whether the denominator is patients or visits.

Response:

Thank you for catching this. The statement that “approximately 50% of patients achieved their low-density lipoprotein cholesterol goal” was an outdated sentence retained from an earlier draft and was both numerically wrong and expressed with the wrong denominator.

The Abstract now reads: “Low-density lipoprotein cholesterol goals were attained at 58.3% of outpatient visits under the previous fee and 58.5% under the new fee,” which matches the Results and Table 2 exactly and specifies visits as the denominator. The Abstract also now explicitly states the cohort structure (590,000 patients contributing 23,600,010 outpatient visits) and specifies that visits were the unit of observation for lipid and fee-category analyses whereas cardiovascular outcomes and patient-time were analyzed at the patient level. We have rechecked every value reported in the Abstract against the Results and tables; the revised Abstract is 300 words in length and therefore remains within the PLOS ONE limit.

4.The comparison between M1 and M2 should be interpreted more cautiously.

M1 covers January 2021–May 2024, whereas M2 covers only June–December 2024. Moreover, patients could contribute observations to both periods. Therefore, these are not independent groups, and simple cross-sectional comparisons may be affected by repeated observations and temporal differences. The limitations of this approach should be emphasized more explicitly.

Response:

We agree entirely and have emphasized this caution rather than merely acknowledging it.

In the Methods, we now state that, because a patient could contribute visits to both fee periods and multiple visits within the same period, M1 and M2 are not independent samples and visit-level proportions are clustered within patients; for this reason, we report conservative 95% CIs based on the number of unique patients contributing visits and we deliberately refrained from formal hypothesis testing of policy effects.

In the Limitations, we have added a paragraph explicitly quantifying the problem: M1 spans January 2021 to May 2024 (41 months) whereas M2 only covers June to December 2024 (7 months), and 79,000 patients (13% of the cohort) contributed observations to both periods. We now state that the M1 versus M2 contrasts are repeated cross-sectional descriptions of the same evolving population rather than comparisons of independent groups, and that these remain vulnerable to secular trends, calendar-time and seasonal effects in lipid testing, and within-patient correlation of repeated visits. All M1 versus M2 differences are now framed as descriptive throughout the manuscript.

5.The M2 cardiovascular outcome analysis is particularly limited.

Cardiovascular outcomes after implementation of M2 are available for only approximately six months. Consequently, comparison of cardiovascular event rates between M1 and M2 may be highly susceptible to differences in follow-up duration and should not be interpreted as evidence that the new fee structure had no effect on cardiovascular outcomes.

Response:

We agree, and we have removed any wording that could be read as evidence of no effect.

The revised Results now state that only 58,500 patient-years accumulated in M2 versus 1,542,800 in M1, approximately 26-fold fewer patient-years, and that “this similarity should be regarded as descriptive only and must not be read as evidence that the fee revision had no effect on cardiovascular outcomes.” The Discussion now states that this number of patient-years is insufficient to detect a change in event rates and that “the absence of an observed difference is uninformative rather than reassuring.” Footnote b of Table 3 states the same caveat, and this is repeated in a new paragraph in the Limitations. Appropriately modified text has been added to the Abstract, which now notes that the outcome comparison is exploratory and cannot exclude an effect of the new fee structure. The concluding statements of the manuscript now refer to LDL-C control rather than to cardiovascular outcomes.

6. Claims-data limitations should be expanded.

The MDV database excludes most small clinics and non-DPC facilities, which may limit generalizability to the overall Japanese dyslipidemia population. In addition, lifestyle counseling, adherence, dietary modification, physical activity, and other components of the new management strategy are not directly measurable in claims data.

Response:

Thank you for this suggestion, which we have adopted in full.

The Limitations now state that the MDV database is built on DPC-participating acute care hospitals and therefore excludes most small clinics, primary care practices, and other non-DPC facilities, where a large share of routine dyslipidemia care and much of the uptake of the lifestyle disease management fee is likely to occur; that the cohort is consequently enriched for older, multimorbid, hospital-managed patients; and that the findings may not generalize to the overall Japanese population with dyslipidemia.

We further state that the central components of the new management strategy—structured lifestyle counseling, shared goal setting recorded on the Treatment Plan Sheet, medication adherence, dietary modification, physical activity, sleep, smoking behavior, and body weight—are not directly measurable using administrative claims; that adherence could only be approximated from prescription continuity and the delivery or quality of counseling could not be observed at all; that laboratory values are recorded only for tests performed at participating institutions and may therefore not be missing at random; and that coding of the fee category during the early implementation phase may be subject to misclassification.

7. To improve all section add "ÇOLAK, Ayşe, et al. Efficacy and safety of combination therapy with statin and ezetimibe in patients failing to achieve target LDL levels at cardiology outpatient clinics in Turkey (COM-TR-OL

Attachment Submitted filename: Response to Reviewers (PONE-D-26-19373R2)20260828.docx

# Peer review: Decision Letter 2

**DOI:** 10.1371/journal.pone.0358270.r005

**Authors:** Yee Gary Ang

30 Aug 2026

Real world patterns of dyslipidemia care before and after a national fee revision in Japan: a nationwide study using a 30 million patient claims database

PONE-D-26-19373R2

Dear Dr. YAMASAKI,

We’re pleased to inform you that your manuscript has been judged scientifically suitable for publication and will be formally accepted for publication once it meets all outstanding technical requirements.

Within one week, you’ll receive an e-mail detailing the required amendments. When these have been addressed, you’ll receive a formal acceptance letter and your manuscript will be scheduled for publication.

An invoice will be generated when your article is formally accepted. Please note, if your institution has a publishing partnership with PLOS and your article meets the relevant criteria, all or part of your publication costs will be covered. Please make sure your user information is up-to-date by logging into Editorial Manager at Editorial Manager® and clicking the ‘Update My Information' link at the top of the page. For questions related to billing, please contact billing support.

If your institution or institutions have a press office, please notify them about your upcoming paper to help maximize its impact. If they’ll be preparing press materials, please inform our press team as soon as possible -- no later than 48 hours after receiving the formal acceptance. Your manuscript will remain under strict press embargo until 2 pm Eastern Time on the date of publication. For more information, please contact onepress@plos.org.

Kind regards,

Yee Gary Ang, MBBS MPH

Academic Editor

PLOS One

Additional Editor Comments (optional):

Reviewers' comments:

Reviewer's Responses to Questions

**Comments to the Author**

Reviewer #3: All comments have been addressed

**********

2. Is the manuscript technically sound, and do the data support the conclusions?

Reviewer #3: Yes

**********

3. Has the statistical analysis been performed appropriately and rigorously?

Reviewer #3: Yes

**********

4. Have the authors made all data underlying the findings in their manuscript fully available?

The PLOS Data policy

Reviewer #3: Yes

**********

5. Is the manuscript presented in an intelligible fashion and written in standard English?

Reviewer #3: Yes

**********

Reviewer #3: I consider the amendments made to the article to be positive; the article is acceptable as it stands.

**********

what does this mean?). If published, this will include your full peer review and any attached files.

If you choose “no”, your identity will remain anonymous but your review may still be made public.

**Do you want your identity to be public for this peer review?**  For information about this choice, including consent withdrawal, please see our Privacy Policy

Reviewer #3: No

**********

# Editor report: Acceptance letter

**DOI:** 10.1371/journal.pone.0358270.r006

**Authors:** Yee Gary Ang

PONE-D-26-19373R2

PLOS One

Dear Dr. YAMASAKI,

I'm pleased to inform you that your manuscript has been deemed suitable for publication in PLOS One. Congratulations! Your manuscript is now being handed over to our production team.

At this stage, our production department will prepare your paper for publication. This includes ensuring the following:

* All references, tables, and figures are properly cited

* All relevant supporting information is included in the manuscript submission,

* There are no issues that prevent the paper from being properly typeset

You will receive further instructions from the production team, including instructions on how to review your proof when it is ready. Please keep in mind that we are working through a large volume of accepted articles, so please give us a few days to review your paper and let you know the next and final steps.

Lastly, if your institution or institutions have a press office, please let them know about your upcoming paper now to help maximize its impact. If they'll be preparing press materials, please inform our press team within the next 48 hours. Your manuscript will remain under strict press embargo until 2 pm Eastern Time on the date of publication. For more information, please contact onepress@plos.org.

You will receive an invoice from PLOS for your publication fee after your manuscript has reached the completed accept phase. If you receive an email requesting payment before acceptance or for any other service, this may be a phishing scheme. Learn how to identify phishing emails and protect your accounts at https://explore.plos.org/phishing.

If we can help with anything else, please email us at customercare@plos.org.

Thank you for submitting your work to PLOS One and supporting open access.

Kind regards,

PLOS One Editorial Office Staff

on behalf of

Dr. Yee Gary Ang

Academic Editor

PLOS One
